# Targeting Ca^2 +^ Handling Proteins for the Treatment of Heart Failure and Arrhythmias

**DOI:** 10.3389/fphys.2020.01068

**Published:** 2020-09-04

**Authors:** Alexandra Njegic, Claire Wilson, Elizabeth J. Cartwright

**Affiliations:** ^1^Division of Cardiovascular Sciences, The University of Manchester, Manchester, United Kingdom; ^2^Centre for Tumour Biology, Barts Cancer Institute, Queen Mary University of London, London, United Kingdom; ^3^Institute of Translational Medicine, University of Liverpool, Liverpool, United Kingdom

**Keywords:** cardiovascular disease, heart failure, arrhythmia, calcium dysregulation, calcium

## Abstract

Diseases of the heart, such as heart failure and cardiac arrhythmias, are a growing socio-economic burden. Calcium (Ca^2+^) dysregulation is key hallmark of the failing myocardium and has long been touted as a potential therapeutic target in the treatment of a variety of cardiovascular diseases (CVD). In the heart, Ca^2+^ is essential for maintaining normal cardiac function through the generation of the cardiac action potential and its involvement in excitation contraction coupling. As such, the proteins which regulate Ca^2+^ cycling and signaling play a vital role in maintaining Ca^2+^ homeostasis. Changes to the expression levels and function of Ca^2+^-channels, pumps and associated intracellular handling proteins contribute to altered Ca^2+^ homeostasis in CVD. The remodeling of Ca^2+^-handling proteins therefore results in impaired Ca^2+^ cycling, Ca^2+^ leak from the sarcoplasmic reticulum and reduced Ca^2+^ clearance, all of which contributes to increased intracellular Ca^2+^. Currently, approved treatments for targeting Ca^2+^ handling dysfunction in CVD are focused on Ca^2+^ channel blockers. However, whilst Ca^2+^ channel blockers have been successful in the treatment of some arrhythmic disorders, they are not universally prescribed to heart failure patients owing to their ability to depress cardiac function. Despite the progress in CVD treatments, there remains a clear need for novel therapeutic approaches which are able to reverse pathophysiology associated with heart failure and arrhythmias. Given that heart failure and cardiac arrhythmias are closely associated with altered Ca^2+^ homeostasis, this review will address the molecular changes to proteins associated with both Ca^2+^-handling and -signaling; their potential as novel therapeutic targets will be discussed in the context of pre-clinical and, where available, clinical data.

## Introduction

Cardiovascular diseases, such as ischemic heart disease, stroke, and arrhythmias, are the leading cause of death globally (Roth et al., 2017); with over 17 million fatalities attributed annually to CVD (Collaborators GBDCoD., 2018). Acquired CVD are thought to arise due to a combination of modifiable environmental causes and underlying genetic pre-dispositions (Kannel et al., 1961; Khot et al., 2003). CVDs lead to both functional and structural changes to the vasculature and myocardium. In the heart, cardiac dysfunction encompassing cardiac decompensation, arrhythmogenesis and impaired contractility are all closely associated with changes to calcium (Ca^2+^) handling proteins, Ca^2+^ channels and Ca^2+^ pumps (Lou et al., 2012). Determining the full scope of changes to Ca^2+^-handling proteins and mechanisms will aid in the development of novel therapeutics to reverse and/or prevent CVD. This review will focus on how changes to Ca^2+^-handling proteins observed during cardiac arrhythmogenesis and HF have been therapeutically targeted, with emphasis placed on recent developments in the search for novel therapies.

## Ca^2+^ and Ca^2+^-Handling Proteins in the Heart

### Maintenance of Ca^2+^ Homeostasis

**FIGURE 1:**
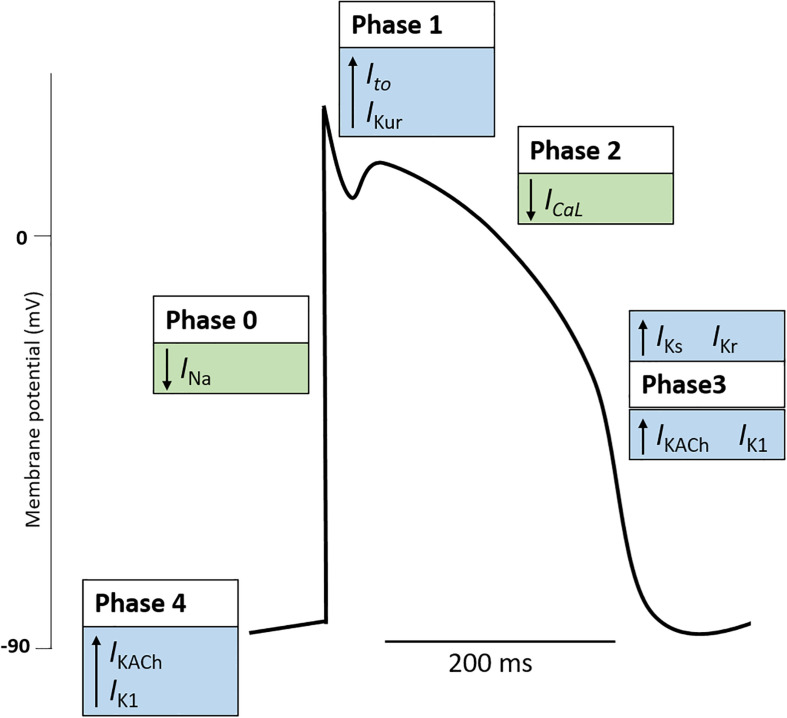
The cardiac action potential. The cardiac action potential represents the electrical activity of a cardiomyocyte during cardiac contraction and is governed by changes in outward (blue boxes) and inward (green boxes) cardiac ion currents. Generally, the cardiac action potential is divided into five Phases. (Phase 4: Resting) The resting membrane potential is stable (∼–90 mV) and maintained by outward leak of K^+^ (*I*_KACh_, *I*_K__1_). Here the Na^+^ and Ca^+^ channels are closed. (Phase 0: Upstroke) An action potential at a neighboring cardiomyocyte initiates a rise in membrane potential. When the membrane potential reaches the threshold potential (∼70 mV), Na^+^ channels open, resulting in a rapid influx of Na^+^ and the generation on the inward Na^+^ current (I_Na_). Na^+^ channels become inactivated soon after opening (Phase 1: Early repolarization). Some K^+^ channels transiently open causing an outward K^+^ current (*I*_to_, *I*_Kur_). This results in a period of rapid repolarization. (Phase 2: Plateau phase) L-type Ca^2+^ channels are open to create a small, constant inward Ca^2+^ current (*I*_CaL_) which triggers excitation contraction coupling resulting in cardiomyocyte contraction. K^+^ currents remain active, although the two currents are electrically balanced and maintain a plateau. (Phase 3: Repolarization) Ca^2+^ channels become gradually inactivated and the outflow of K^+^ exceeds the Ca^2+^ inflow. K^+^ currents (*I*_Ks,_ *I*_Kr_, *I*_KACh_, *I*_K__1_) repolarize the cell and the resting membrane potential is restored. Note, the figure represents the typical ventricular cardiac action potential. The action potential of atrial cells and pacemaker cells (those found in the sinus node, atrioventricular node, and Purkinje fibers) differ. K^+^, potassium; Na^+^, sodium; Ca^2+^, calcium; *I*_KACh_, muscarinic-gated K^+^ current; *I*_K__1_, inward rectifier K^+^ current; *I*_Na_, Na^+^ current; *I*_to_, transient outward K^+^ current; *I*_Kur_, delayed rectifier K^+^ current; *I*_CaL_, L-type Ca^2+^ current; *I*_Ks_, delayed rectifier (slow) K^+^ current; *I*_kr_, delayed rectifier (fast) K^+^ current.

The initiation and control of each heartbeat is achieved by the cardiac conduction system consisting of the SAN, AVN, and His-Purkinje system. Electrical impulses, termed cardiac AP arise from the SAN, often referred to as the pacemaker of heart, and propagate throughout the heart (Boyett, 2009). During the cardiac AP, the electrical membrane potential rapidly rises and falls, referred to as depolarization and repolarization respectively, triggering ECC. The changes in membrane potential are driven by the movement of ions across the cell membrane (Grant, 2009). Generally, the cardiac AP is divided into five phases (Figure 1): (Phase 0) involves rapid depolarization of the membrane potential which is key to the propagation of cardiac impulse in the heart; (Phase 1) is a brief interval of rapid depolarization; (Phase 2) known as the plateau phase of the cardiac AP in which Ca^2+^ enters the cell to trigger ECC; (Phase 3) rapid repolarization restores the membrane potential; and (Phase 4) the membrane potential is stable at the resting potential (Grant, 2009). Following an AP, the cell enters a recovery state termed the refractory period. Here, the cell is unable to initiate an AP which allows the heart to prepare for the next contraction (Ramza et al., 1990). The key ionic currents involved in the generation and propagation of the cardiac AP are the Ca^2+^ (*I*_Ca_), sodium (Na^+^, *I*_Na_), and potassium (K^+^, *I*_K_) currents (Grant, 2009). It is worth noting that different cell types within the cardiac conduction system exhibit specific cardiac AP relating to their function. Differences in ion channel expression results in regional specific APs (Monfredi et al., 2010).

**FIGURE 2:**
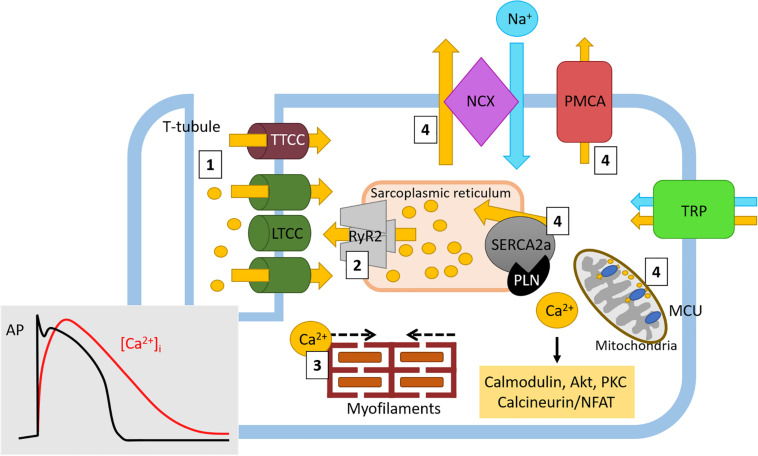
Physiological Ca^2+^ cycling in cardiomyocytes. Ca^2+^ cycling in cardiomyocytes generates cardiac contraction through excitation-contraction coupling. Briefly, (1) Ca^2+^ (yellow circles and arrows) rapidly enters the cell through LTCC where it causes (2) Ca^2+^-induced Ca^2+^ release from Ca^2+^ stores in the sarcoplasmic reticulum. During these early stages of ECC, intracellular Ca^2+^ ([Ca^2+^]_i_) levels rapidly increase (red line, insert) following the onset of the cardiac action potential and cardiomyocyte depolarization (and black line, insert). (3) The amplified Ca^2+^ signal then induces actin-myosin myofilament crossbridge formation, resulting in cardiac contraction. Following this action, as the cardiac action potential enters the plateau phase (black line), the inward [Ca^2+^]_i_ levels begin to slowly decrease (red line). (4) Ca^2+^ is either returned to internal stores through SR-located SERCA2a and mitochondrial MCU or to the extracellular space through NCX (in exchange for 3Na^+^, blue circle and arrow) and PMCA. Here, as cardiac repolarization occurs (black line) the levels of [Ca^2+^]_i_ return to baseline (red line) in order to return membrane polarization to baseline. Free Ca^2+^ can also drive intracellular signaling (yellow box) which can influence gene transcription. Ca^2+^ homeostasis is also maintained by the TRP channel family of proteins. Ca^2+^, calcium; Na^+^, sodium; LTCC, L-type Ca^2+^ channel; TTCC, T-type Ca^2+^ channel; NCX, sodium calcium exchanger; PMCA, plasma membrane Ca^2+^ ATPase; TRP, transient receptor potential (canonical, vallinoid-related, melastatin-related), RyR, ryanodine receptor; SERCA, sarco/endoplasmic reticulum Ca^2+^-ATPase; PLN, phospholamban; MCU, mitochondrial Ca^2+^ uniporter.

In the heart, Ca^2+^ has an indispensable role in cardiomyocyte ECC; in addition, as a signaling molecule, Ca^2+^ also acts as an intracellular second messenger in cardiomyocytes and other cell types. ECC results in cardiac contraction and helps to maintain diastolic and systolic cardiac function (Lou et al., 2012). During ECC, four steps involving Ca^2+^ trigger cardiomyocyte contraction: (1) membrane depolarization causes a rapid influx of Ca^2+^ through the LTCC located on transverse (T)-tubules, which results in an intracellular Ca^2+^ gradient in the junctional zone; (2) Ca^2+^ diffusion activates RyR located on the SR resulting in Ca^2+^ sparks and an amplification of the Ca^2+^ signal in a process termed Ca^2+^-induced Ca^2+^ release; (3) Ca^2+^ released via RyR from internal SR stores binds to troponin-C to cause contraction through actin-myosin filament crossbridge formation; (4) Ca^2+^ is transported out of the cardiomyocyte through NCX and PMCA or back into internal stores through SERCA and MCU (Bers, 2002; Lou et al., 2012) (Figure 2). The function of SERCA is tightly regulated by PLN. In its dephosphorylated form, PLN inhibits SERCA2a but when its phosphorylated at Ser10, Ser16 or Thr17, the inhibition of SERCA2 is relieved (Mattiazzi et al., 2005). Such is the importance of PLN-mediated SERCA2a regulation that PLN ablation induces a hypercontractile cardiac phenotype (Luo et al., 1994). Ca^2+^ homeostasis is also maintained through additional Ca^2+^ channels located on the plasma membrane (Figure 2). The transient receptor potential channels (TRP) are a large family of non-selective cation permeable channels which, depending on the family, permit the influx of Ca^2+^, along with Mg^2+^, Zn^2+^, and Na^+^(Falcon et al., 2019). The TRP family can be further divided into subfamilies, including TRP-canonical (TRPC), TRP vallinoid-related (TRPV), and TRP melastatin-related (TRPM) (Freichel et al., 2017). The ECC Ca^2+^ cycle is tightly regulated by protein kinases and accessory proteins which modify the activity and expression of ECC Ca^2+^ handling proteins. Functional changes to proteins involved in ECC and Ca^2+^ homeostasis can impair contractility and contribute to cardiac dysfunction. Furthermore, changes to Ca^2+^ handling can also impair cardiac conductivity, causing fatal arrhythmias. Altered Ca^2+^ homeostasis can result in abnormal automaticity, triggered activity, and can provide a substrate for re-entry arrhythmias (Landstrom et al., 2017). The contributions these Ca^2+^ handling proteins make to the pathophysiology of cardiac hypertrophy, HF, and cardiac arrhythmias are discussed in detail later in this review.

### Current Therapeutic Approaches Targeting HF and Cardiac Arrhythmia and Their Impact on Ca^2+^-Handling Proteins

Treatment of HF and cardiac arrhythmias can be surgical (including implantation of a pacemaker or implantable cardioverter defibrillator) and/or pharmacological. Primary drug-based therapies for these disorders include targeting (1) the RAAS signaling system, (2) β-adrenergic hyperactivation, and (3) aberrant ion channel activation (Ca^2+^, Na^+^, and K^+^). As the focus of this review concerns how Ca^2+^ handling proteins present a novel therapeutic approach, we discuss the current pharmacological therapies mentioned above in brief, including any known impact they have on Ca^2+^ handling in the heart.

#### The Renin–Angiotensin–Aldosterone System

Renin–angiotensin–aldosterone signaling is elevated in patients with HF and reduced ejection fraction (HFrEF, ejection fraction < 40%) (Watkins et al., 1976) and in advanced HF, the levels of plasma renin activity and aldosterone are increased (Dzau et al., 1981). Furthermore, aldosterone secretion is enhanced in response to ANG-II signaling and its receptor is upregulated in cardiomyocytes from HFrEF patients (Yoshida et al., 2005). Aldosterone alone is pro-hypertrophic, pro-fibrotic and pro-inflammatory (Pinilla-Vera et al., 2019). Increased neurohormonal signaling can activate the MAPK which, in turn, upregulates pro-hypertrophic gene transcription (Zhang et al., 2003; Nakamura and Sadoshima, 2018). The MAPK signaling cascade comprises four main ‘typical’ branches; ERK1/2, JNK, p38 and ERK5, all with distinct upstream regulators (Rose et al., 2010) and activation of the ERK1/2 signaling cascade may determine whether concentric or eccentric cardiomyocyte growth and remodeling occurs (Bueno et al., 2000; Lorenz et al., 2009; Kehat et al., 2011). Activation of RAAS can also promote cardiac arrhythmias including AF and ventricular tachyarrhythmias, partly due to the formation of a pro-arrhythmia substrate in cardiac hypertrophy and HF. In addition, RAAS is also known to directly affect cardiac ion channels. Angiotensin and aldosterone have been shown to attenuate the transient outward K^+^ current (*I*_to_) and enhance *I*_CaL_, impacting AP duration and repolarization. Whilst oxidative stress triggered by RAAS activation is reported to affect cardiac ion currents and several Ca^2+^ handling proteins (Iravanian and Dudley, 2008).

The maladaptive effects associated with over-activation of the RAAS is one of main focuses of current therapeutics; both ACEI and ARBs are prescribed to patients with HF to help improve cardiac function and survival (McMurray, 2004; Demers et al., 2005). Several clinical trials have shown that ACEI and ARBs reduce mortality in patients with HFrEF (Kjekshus et al., 1992; Maggioni et al., 2002; McMurray et al., 2003). In addition, further inhibition of RAAS is achieved through aldosterone antagonism. Aldosterone receptor blockade with an agent such as eplerenone has been shown to reduce the risk of death and hospitalization events in patients with HFrEF (Pitt et al., 2003; Zannad et al., 2011). However, the absolute beneficial use of aldosterone agonists in patients with HF and preserved ejection fraction (HFpEF, ejection fraction > 50%) is still yet to be determined (Pitt et al., 2014). Studies have also aimed to dissect the use of RAAS inhibitors in patients with cardiac arrhythmias (Iravanian and Dudley, 2008). ACEI and ARBs have been shown to reduce the recurrence rate in patients with existing AF (Madrid et al., 2002; Ueng et al., 2003), and reduce the incidence of new onset AF in hypertensive (Wachtell et al., 2005)- and post-MI (Pedersen et al., 1999) patients. Treatment with ACEI has also been associated with a reduced risk of sudden cardiac death in HF patients (Heart Outcomes Prevention Evaluation Study Investigator, Yusuf et al., 2000).

#### The β-Adrenergic Pathway

In HF, elevated sympathetic activity increases blood catecholamines; an increase which inversely correlates with survival (Lymperopoulos et al., 2013). The initial elevation in sympathetic activation is beneficial as cardiac contractility is enhanced to maintain function; however, chronic activation of β-adrenergic receptors (β-AR) promotes cardiac dysfunction through increased cardiomyocyte hypertrophy (Lohse et al., 2003). Catecholamine binding to β-AR activates AC and subsequently leads to an increase in cAMP levels; cAMP activates PKA which phosphorylates proteins associated with Ca^2+^ handling, including LTCC (Schroder et al., 1998), RyR2 (Wehrens et al., 2006), and PLN (Antos et al., 2001; Nakamura and Sadoshima, 2018), together culminating in increased levels of cytosolic Ca^2+^ (Port and Bristow, 2001; Nakamura and Sadoshima, 2018). In addition to phosphorylation of key Ca^2+^ handling proteins, β-AR signaling also results in the *s*-nitrosylation of many Ca^2+^ handling proteins in cardiomyocytes, such as PLN and cardiac troponin C (Irie et al., 2015). Furthermore, activation of β-AR is the primary mechanism underlying sympathetic control of heart rhythm, with many of the cardiac ionic currents responsive to β-adrenergic stimulation (Gardner et al., 2016). Unsurprisingly, enhanced adrenergic activity has been identified in arrhythmic conditions, including AF, and results in enhanced automaticity and triggered activity (Workman, 2010; Antzelevitch and Burashnikov, 2011).

Therapeutic targeting of this excessive β-adrenergic response is achieved using β-blockers. β-blockers are deployed to improve cardiac function, reduce incidence of arrhythmias, and decrease mortality associated with HF by diminishing the hyperactivity of the β-adrenergic pathway. Improvements in mortality rates in patients with HFrEF have been recorded, irrespective of the type of β-blocker (Chatterjee et al., 2013; Pinilla-Vera et al., 2019), and large randomized trials have provided further evidence of the advantageous effects of single β-blockers agents in the treatment of HFrEF (CIBIS-II Investigators and Committees, 1999; Tepper, 1999; Packer et al., 2002). Furthermore, a meta-analysis of all clinical trials involving the use of β-blockers in HFpEF patients suggested the use of β-blockers may reduce mortality by 22% (Fukuta et al., 2017). Interestingly, through their mechanism of action, β-blockers also inhibit PKA hyperphosphorylation of the RyR2 channel in both human and animal models of HF (Reiken et al., 2001, 2003); the proposed mechanism by which β-blockers improve cardiac function in HF is by preventing PKA hyperphosphorylation of RyR2 Ser2808 and subsequent receptor destabilization (Shan et al., 2010). β-blockers also display multiple antiarrhythmic effects including a lowering of heart rate, a decrease in SAN/AVN automaticity and re-entry events, and a reduction in afterdepolarizations. Notably, β-blockers are used for the treatment of sinus and supraventricular tachycardias and in rate control of AF and ventricular tachyarrhythmias (Lei et al., 2018). In addition, β-blockers are also used to treat some patients with LQTS, thus reducing the risk of adverse cardiac events; although it is reported that some β-blockers are more effective than others (Chockalingam et al., 2012; Abu-Zeitone et al., 2014). Patients with CPVT are prescribed β1/β2-blockers such as nadolol which, when compared to β1-selective blockers, reduces the severity and incidence of ventricular arrhythmias (Leren et al., 2016).

#### Ion Channel Blockers; Ca^2+^, Na^+^, and K^+^

The voltage activated LTCC is a multi-subunit protein which, under basal conditions, provides the primary entry point for rapid Ca^2+^ influx. During HF, increased phosphorylation of LTCCs may result in redistribution of LTCCs and increased channel open probability (Schroder et al., 1998; Sanchez-Alonso et al., 2016) which can lead to increased Ca^2+^ influx, a phenomenon implicated in both hypertrophy and HF (Richard et al., 1998). Changes in LTCC dynamics have also been associated with the development of arrhythmias, and numerous LTCC mutations have been identified in primary arrhythmic disorders such as LQTS and Brugada syndrome (Grant, 2009). The LTCC is the primary target of Ca^2+^ channel blockers which are currently extensively used to treat hypertension and certain arrhythmic disorders, but are used with caution in HF. Inhibiting the LTCC with amlodipine prevents adverse cardiac remodeling in a mouse model of TAC-induced pressure overload hypertrophy (Liao et al., 2005). However, cardiac-specific knockdown of LTCC has the opposing effect and results in pathological hypertrophy partly due to a compensatory increase in Ca^2+^ leak from the SR via RyR2 (Goonasekera et al., 2012).

Ca^2+^ channel blockers (non-dihydropyridines and dihydropyridines) are generally not prescribed to patients with HFrEF, except for amlodipine. Due to their negative inotropic effects, clinical studies have demonstrated that non-dihydropyridines increase mortality rates and incidence of late-onset HF (The Danish Study Group on Verapamil in Myocardial Infarction, 1990; Goldstein et al., 1991) and should be avoided to prevent further depression of cardiac function. However, amlodipine (a dihydropyridine) does not affect mortality so can be used to treat HFrEF patients with angina and/or hypertension (Packer et al., 1996). On the other hand, both classes of Ca^2+^ channel blockers have no adverse effects on mortality or HF hospitalization in patients with HFpEF (Patel et al., 2014); although, non-dihydropyridines tend only to be considered when β-blockers alone are unsuccessful (Godfraind, 2017).

Ca^2+^ channel blockers are currently approved antiarrhythmic agents used to treat arrhythmias arising from SAN and AVN dysfunction; the AP generated by these areas of the heart are strongly dependent on Ca^2+^ entry (Lei et al., 2018). The non-dihydropyridines Ca^2+^ channel blockers used for the treatment of arrhythmias (supraventricular arrhythmias, ventricular tachycardias occurring in the absence of structural heart disease, and AF) include phenylalkylamines and benzothiazepines (for example, verapamil, and diltiazem, respectively) (January et al., 2014; Lei et al., 2018). However, as noted above, non-dihydropyridines should not be used in patients with decompensated HF. In addition, non-dihydropyridines should be avoided in patients with AF combined with pre-excitation (a condition in which the ventricles activate too early) as treatment may increase the risk of ventricular fibrillation (January et al., 2014). There is evidence that combining Ca^2+^ channel blockers with other pharmacological approaches can enhance the treatment of arrhythmic conditions. For example, in patients with CPVT, the addition verapamil to existing β-blocker therapy was shown to reduce the incidence of exercise-induced arrhythmias when compared to β-blocker therapy alone (Rosso et al., 2007).

Na^+^ channel blockers target the voltage-gated Na^+^ channel, Na_v_1.5, which drives the inward Na^+^ current responsible for the rapid depolarization at the start of the cardiac AP (Lei et al., 2018). The aim of Na^+^ channel blockers is therefore to reduce the rate of the AP upstroke and reduce AP conduction in the atria, ventricle, and Purkinje fiber tissue (where Na_v_1.5 is preferentially expressed). Class I Na^+^ channel blockers are further subclassified based on their molecular properties and interaction with the Na_v_1.5 (Lei et al., 2018; Li et al., 2019). Class Ia (quinidine, ajmaline, disopyramide) and Ib (lidocaine, mexiletine) Na^+^ channel blockers are used to treat patients with sustained ventricular tachycardia and ventricular fibrillation. In addition, quinidine has been indicated for short QT syndrome and Brugada syndrome and mexiletine suggested for the treatment of premature ventricular complexes, LQT3, and ventricular arrhythmias after MI (Al-Khatib et al., 2018; Lei et al., 2018). Class Ic agents (flecainide, propafenone) are used for ventricular tachycardia, premature ventricular complexes, and AF, with flecainide also having a role in the treatment of CPVT (January et al., 2014; Al-Khatib et al., 2018). However, Class Ic agents should not be used in patients with underlying structural heart disease (January et al., 2014). Ranolazine, a Class Id agent, is used to treat ventricular tachycardia and differs from Class Ia-Ic agents by inhibiting the late Na^+^ current (Al-Khatib et al., 2018; Lei et al., 2018). Despite Na^+^ channel blockers being identified as an effective antiarrhythmic treatment; they have also been shown to be proarrhythmic. The Cardiac Arrhythmia Suppression Trial (CAST) in the late 1980s studied the effect of Na^+^ channel blockers on patients with ventricular ectopic activity up to 2 years following a MI. The study revealed a higher rate of death in patients receiving flecainide or encainide compared to the placebo group (Cardiac Arrhythmia Suppression Trial I., 1989). Na^+^ channel blockers are therefore generally limited to patients who do not have structural heart disease.

The Class III K^+^ channel blockers work by binding to K^+^ channels involved in Phase 3 repolarization, and thus delay repolarization and increase AP duration (Lei et al., 2018). Class IIIa K^+^ blockers can be divided based on their K^+^ channel target. Non-selective K^+^ channel blockers (ambasilide, amiodarone, dronedarone) and K_v_11.1 blockers (dofetilide, ibutilide, sotalol) have been indicated for the treatment of ventricular tachycardia in patients with structural heart disease or with remote MI, tachyarrhythmias in patients with Wolff-Parkinson-White syndrome, rate control in AF [with amiodarone the most effective antiarrhythmic drug for maintenance of sinus rhythm in patients with persistent or paroxysmal AF (January et al., 2014)], ventricular fibrillation and premature ventricular contraction, and supraventricular tachyarrhythmias (January et al., 2014; Al-Khatib et al., 2018; Lei et al., 2018). Another type of K^+^ channel blocker which targets K_v_1.5 (vernakalant) has been suggested for the treatment of AF (Lei et al., 2018). Similar to Na^+^ channel blockers, K^+^ blockers can exhibit proarrhythmic effects. QT interval prolongation caused by K^+^ channel blockers has been shown to predispose patients to ventricular arrhythmias and TdP (McCauley et al., 2016). Due to the AP prolongation effect, K^+^ channel blockers should not be given to patients with LQTS, those being treated with QT-prolonging drugs, or with comorbidities associated with an increased risk for TdP (Dan et al., 2018). In addition, co-administration of amiodarone with other cardiac therapeutics including β-blockers, can result in bradycardia and atrioventricular block (Dan et al., 2018). As well as K^+^ channel blockers, Class IIIb drugs are metabolically dependent K^+^ channel openers and target K_ir_6.2 channels (although these have not been indicated in the treatment of arrhythmias) (Lei et al., 2018). A recent updated classification determined a Class IIIc group made up of transmitter dependent K^+^ channel blockers with BMS 914392 currently under review for the management of AF (Lei et al., 2018).

## Pathological Cardiac Hypertrophy and Heart Failure

Cardiac hypertrophy occurs under both physiological and pathological conditions. Hypertrophy itself is an adaptive cardiac response which arises due to either pressure- or volume-cardiac overload. Typically, in physiological hypertrophy, the increase in cardiomyocyte size is initially due to the addition of sarcomeres in parallel (eccentric hypertrophy) which increases both ventricular volume and relative wall thickness, resulting in increased contractility which preserves cardiac output (Nakamura and Sadoshima, 2018). Conversely, in pathological hypertrophy cardiomyocytes are added in series (concentric hypertrophy) in order to compensate for the increased cardiac stress, this type of hypertrophy results in maintained or reduced ventricle volume but an increased relative wall thickness (Sawada and Kawamura, 1991; Lazzeroni et al., 2016; Nakamura and Sadoshima, 2018). Furthermore, persistent maladaptive remodeling can eventually lead to ventricular dilatation (eccentric hypertrophy), contractile dysfunction and impaired cardiac output (Sawada and Kawamura, 1991; Lazzeroni et al., 2016; Nakamura and Sadoshima, 2018).

It is now widely believed that each form of hypertrophy is regulated by distinct molecular mechanisms that drive the differing cardiac phenotypes and overall prognosis. Despite the activation of initial compensatory hypertrophic mechanisms, maladaptive remodeling occurs when the following processes occur: cell death (apoptosis, necrosis, autophagy), deposition of fibrosis throughout the myocardium, impaired mitochondrial function and regulation, dysregulation of Ca^2+^-handling proteins, re-expression of fetal genes, changes to sarcomere structure and rarefaction of the cardiac microvasculature (Nakamura and Sadoshima, 2018). Chronic maladaptive remodeling witnessed during the pathological response to hypertrophy often develops into HF with the diagnosis of HF itself rapidly becoming a universal healthcare and financial burden. HF is an end-stage CVD which frequently occurs secondary to many different aetiologies (Ziaeian and Fonarow, 2016). Physiologically, HF can be defined as (1) insufficient cardiac output to meet metabolic demands or (2) adequate cardiac output with increased left ventricular filling pressure through activation of compensatory neurohormonal pathways (Ponikowski et al., 2016). HF can be further classified into one of three subtypes: HFpEF, HFrEF or HFmrEF) (Ponikowski et al., 2016; Rastogi et al., 2017). These subclassifications are divided based on quantitation of ejection fraction; HFrEF < 40%, HFmrEF > 40% but < 50% and HFpEF > 50% (Ponikowski et al., 2016; Ziaeian and Fonarow, 2016; Rastogi et al., 2017).

The presence of altered Ca^2+^ transients in HF has been well-documented since the late 1980s. These early studies were amongst the first to show that cardiomyocytes isolated from the failing human heart exhibit reduced Ca^2+^ transient amplitudes, fewer actin-myosin crossbridge formations, a slower rate of Ca^2+^ removal and increased resting intracellular Ca^2+^ when compared to healthy cardiomyocytes (Gwathmey et al., 1987, 1991; Beuckelmann et al., 1992; Hasenfuss et al., 1993). In broad terms, the failing cardiomyocyte shows impaired Ca^2+^ cycling, Ca^2+^ release from the SR and Ca^2+^ removal. Therefore, altering Ca^2+^ in HF through modulation of Ca^2+^ handling proteins has long been investigated as a therapeutic target.

## Calcium Handling Proteins in Cardiac Hypertrophy and HF and Their Potential as Novel Therapeutic Targets

The National Institute of Clinical Excellence (NICE, United Kingdom) and other regulatory bodies such as the European Society of Cardiology recommends ACEIs and β-blockers as first-line treatment for HF (Al-Mohammad et al., 2010; Ponikowski et al., 2016); however, dysregulation of Ca^2+^ handling proteins occurs exclusively during pathological hypertrophy (and not physiological hypertrophy) which suggests altered Ca^2+^ cycling and signaling precedes Ca^2+^ dysregulation observed in HF. The following section of this review will focus on how proteins associated with Ca^2+^-handling, encompassing Ca^2+^ signaling proteins and Ca^2+^ channels and pumps, are altered in cardiac hypertrophy and HF.

### Ca^2+^ Homeostasis and Signaling in Hypertrophy and HF

**FIGURE 3:**
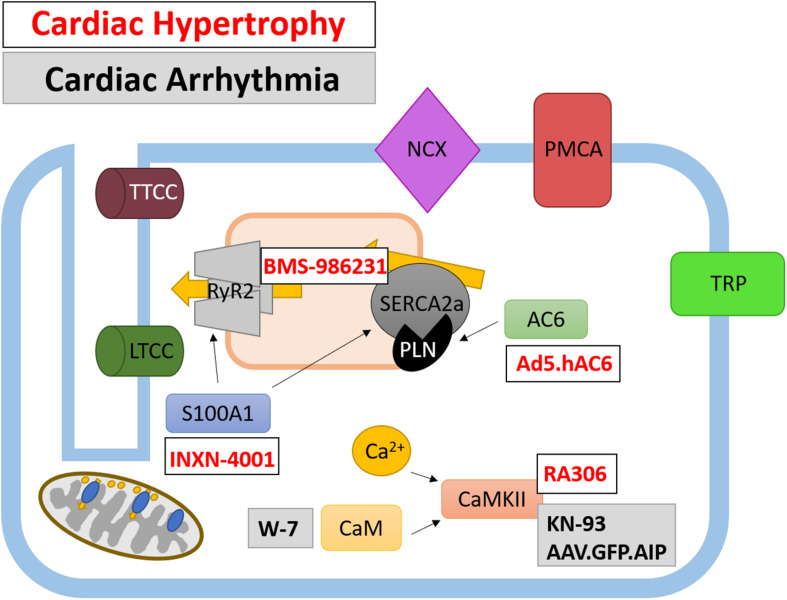
Therapeutically targeted Ca^2+^-handling proteins and their function in cardiomyocytes. Ca^2+^ handling proteins are extensively involved in intracellular signaling and the regulation of Ca^2+^-channels and pumps, as such their modulation presents a different avenue for therapeutic targeting. Both current and potential therapeutics are shown for cardiac hypertrophy (white box, red text) and cardiac arrhythmias (gray box, black text).

Given the importance of Ca^2+^-dependent signaling pathways in the development of hypertrophy and HF, there is scope for developing therapies which target their aberrant expression and activity. Three such potential targets are calmodulin (CaM), calcineurin and CaMKII which are all activated following the release of Ca^2+^ from internal stores. CaMKII signaling is upregulated in human HF (Anderson, 2009). Evidence from animal studies suggests that CaMKII-δ is important in the cardiac stress response as CaMKII-δ null mice show no overt cardiac phenotype under physiological conditions but have an attenuated response to pressure-overload induced hypertrophy (Backs et al., 2009; Ling et al., 2009). CaMKII-δ may influence cardiac hypertrophy through two distinct mechanisms which are mediated by different splice variants; CaMKII-δ_C_ regulates phosphorylation of RyR and PLN resulting in increased Ca^2+^ spark frequency and decreased SR Ca^2+^ content whereas both CaMKII-δ_C_ and CaMKII-δ_B_ variants mediate pro-hypertrophic gene transcription (Zhang et al., 2007). Despite this data to suggest CaMKII plays an important role in HF, no clinically available compounds are currently in clinical trials. However, a recently developed, novel ATP-competitive CaMKII-δ and CaMKII-γ inhibitor, RA306, has shown potential in animal studies (Beauverger et al., 2020) (Figure 3 and Table 1). RA306 prevented phosphorylation of PLN (Thr17) *in vitro* and *in vivo* following stimulation with isoproterenol (Beauverger et al., 2020). Furthermore, twice-daily RA306 treatment was sufficient to increase ejection fraction in a mouse model of dilated cardiomyopathy; importantly, the study used aged mice with advanced cardiac disease to demonstrate the ‘curative’ ability of RA306 (Beauverger et al., 2020).

**TABLE 1:** Therapeutic targeting of Ca^2+^-handling proteins in cardiac hypertrophy, HF and arrhythmia.

**Target**	**Pre-clinical**	**FDA approved**	**Clinical trial identifier**	**Status**	**Results**
S100A1	INXN-4001		NCT03409627	A	N/A
CaM	W-7		Pre-clinical	N/A	N/A
CaMKII	RA306		Pre-clinical	N/A	N/A
	KN-93		Pre-clinical	N/A	N/A
	AAV.GFP.AIP		Pre-clinical	N/A	N/A
AC6	Ad5.hAC6		NCT00787059	C	Safe, tolerable (Hammond et al., 2016)
			NCT03360448	W	N/A
PLN	BMS-986231		NCT03016325	C	Safe, well-tolerated; adverse effects included headaches (Cowart et al., 2019)
			NCT02157506	C	Increased treatment-emergent adverse events in treatment group (>55% in all three regimes vs. 25% in placebo)
			NCT03016325	C	None posted
			NCT03357731	C	None posted
			NCT03730961	C	None posted

*For each therapy listed in Figure 3, their status as either pre-clinical or FDA-approved is listed in the table. In addition, any clinical trial information available for the therapies listed in the table is also presented, where NCT denotes the number given to clinical trials on clinicaltrials.gov. Therapies which have yet to enter registered clinical trials are shown as pre-clinical. A brief description on the in human safety and tolerability of each therapy is also shown where available. A, active; C, completed; N/A, not applicable; W, withdrawn.*

The use of gene therapy to treat CVD has garnered much attention in recent years and has been used as a tool to alter expression of a wide variety of Ca^2+^-handling proteins. Recent work has been undertaken to trial a triple gene plasmid construct INXN-4001 in patients with an outpatient left ventricular assist device [clinicaltrials.gov Identifier NCT03409627] (Figure 3 and Table 1). Plasmid INXN-4001 contains human VEGF165, S100A1 and stromal cell-derived factor 1; expression of these three proteins has been designed to target fibrosis, angiogenesis and impaired cardiac contractility (Jaruga-Killeen et al., 2019). To date there are no reported adverse cardiac-specific safety effects after one infusion of INXN-4001 (Jaruga-Killeen et al., 2019). Of interest to this review is the expression of constitutively active S100A1 protein within the INXN-4001 plasmid. S100A1 is a Ca^2+^ binding protein which controls Ca^2+^-dependent networks in cardiomyocytes through, for example, modulation of RyR2 and SERCA2a activity (Ritterhoff and Most, 2012). S100A1 protein levels are reduced in cardiomyocytes from both human and animal model-derived failing myocardium and levels of reduced S100A1 are associated with poorer contractile function (Du et al., 2002; Most et al., 2006). Adenoviral-based delivery of S100A1 is able to rescue cardiac function in mouse, rat, and pig models of MI-induced HF (Most et al., 2004; Pleger et al., 2005, 2007, 2011; Kraus et al., 2009). Importantly, S100A1 gene transfer alone can restore Ca^2+^ homeostasis in acute and chronic models of HF (Most et al., 2004; Pleger et al., 2005, 2007, 2011; Kraus et al., 2009). However, gene therapy using S100A1 alone has not yet been developed for use in clinical trials.

Gene therapy has also been utilized to overexpress AC6 (Kieserman et al., 2019) (Figure 3 and Table 1). The primary role of AC6 is to catalyze the conversion of ATP to cAMP; however, AC6 can also downregulate the expression of PLN, independently of cAMP concentration (Gao et al., 2004). Adenoviral transfer of AC6 (Ad.AC_VI_) in a porcine model of congestive HF successfully improved LV function (Lai et al., 2004). The promising outcome of Ad.AC_VI_ treatment in a large animal models of HF led to its development for a first in man clinical trial (Ad5.hAC6, also known as RT-100), beginning in 2010 (Hammond et al., 2016; Penny et al., 2018). Results from this placebo-controlled, randomized clinical trial of 56 HFrEF patients confirmed the safety of Ad5.hAC6 [clinicaltrials.gov Identifier NCT00787059] (Hammond et al., 2016). Furthermore, patients receiving the two highest doses (out of five possible different doses) of Ad5.hAC6 showed an acute improvement in EF, assessed 4 weeks post-viral delivery (Hammond et al., 2016). The promising results from this Phase II clinical trial paved the way for the ‘FLOURISH’ trial, a Phase III follow-up [clinicaltrials.gov Identifier NCT03360448] (Penny et al., 2018). However, the FLOURISH study was withdrawn prior to patient recruitment owing to a change of clinical development plans. Therefore, the clinical benefit of Ad5.hAC6 for the treatment of HF still requires investigation in a large, multi-centre clinical trial.

### Ca^2+^ Handling Proteins During Hypertrophy and HF; Ca^2+^ Channels, Pumps and Exchangers

#### LTCC

Currently, LTCC Ca^2+^ blockers are used with caution in patients with HF; however, there may be some therapeutic potential in targeting a subset of LTCC which have been shown to specifically localize to caveolae and do not present as ‘typical’ LTCC which reside on T-tubules (Makarewich et al., 2012). Within caveolae membrane invaginations, Ca^2+^ entry through LTCC may activate pro-hypertrophic calcineurin/NFAT signaling without contributing to ECC (Makarewich et al., 2012). Interestingly, the calcineurin/NFAT signaling axis only contributes to pathological and not physiological hypertrophy (Wilkins et al., 2004). Inhibition of LTCC with channel blocking Rem proteins specifically targeted to Cav-3 caveolae membranes attenuates the inward Ca^2+^ current and prevents pro-hypertrophic gene activation without affecting contractility of feline cardiomyocytes (Makarewich et al., 2012). However, these findings were not replicated a murine model of cardiac hypertrophy *in vivo* (Correll et al., 2017). Therefore, the direct contribution of LTCC to the development of cardiac hypertrophy remains inconclusive and requires further investigation in different pre-clinical models of HF.

#### SERCA2

In failing human cardiomyocytes and in animal models of hypertrophy and HF, the levels of SERCA2 are reduced (Komuro et al., 1989; Nagai et al., 1989; Mercadier et al., 1990; Lipskaia et al., 2014). Downregulation of SERCA2 and therefore a reduction in SERCA2 activity are concordantly associated with both systolic and diastolic dysfunction (Periasamy et al., 1999). The importance of SERCA2 signaling is evidenced by knockout mouse studies. Cardiomyocyte-specific deletion of SERCA2 in adult mice leads to diastolic dysfunction under basal conditions (Andersson et al., 2009). Furthermore, an important feature of Ca^2+^ dysregulation during cardiac hypertrophy is SR Ca^2+^ leak and at the molecular level, cardiomyocyte loss of SERCA2 results in changes to SR Ca^2+^ handling, characterized by slower cytosolic Ca^2+^ removal, reduced SR content and impaired Ca^2+^ transient magnitude (Andersson et al., 2009; Louch et al., 2010; Li et al., 2012). SERCA2 activity could also be affected in HF due to changes in the phosphorylation status of PLN (Schwinger et al., 1995; Mattiazzi and Kranias, 2014). The reduction in phosphorylation of PLN is partly due to increased activity of calcineurin and protein phosphatase 1, as shown in both human HF and animal models (Neumann et al., 1997; Munch et al., 2002). However, more recent data suggests direct post-transcriptional modifications of SERCA2a contribute to its decreased activity (Kranias and Hajjar, 2012). In HF, SERCA2 can undergo either ‘stimulatory’ modification by glutathionylation on C674 (Adachi et al., 2004) and/or SUMOylation on K480 and K585 (Kho et al., 2011), and ‘inhibitory’ acetylation of K492 (Gorski et al., 2019). These modifications to SERCA2a and its regulatory proteins provides more scope for enhancing SERCA2 activity in HF patients.

**FIGURE 4:**
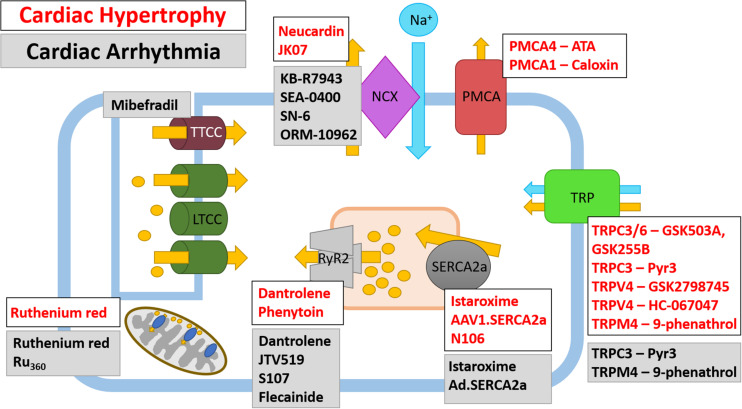
Therapeutic targeting of Ca^2+^ ion channels and pumps in cardiac hypertrophy and cardiac arrhythmia. Ca^2+^ ion channels and Ca^2+^ pumps have been the subject of pharmacological and gene therapy intervention; however, not all therapies have been translated from pre-clinical studies into human trials. Both current and potential therapeutics and their Ca^2+^ ion channel or Ca^2+^ pump target are shown for cardiac hypertrophy (white box, red text) and cardiac arrhythmias (gray box, black text), note there are some agents which have been tested in models of HF and arrhythmia. The LTCC are currently targeted by Ca^2+^ channel blockers which are prescribed to patients with CVD, as such, these are not shown on the schematic.

Restoring SERCA2a protein expression levels has been shown by numerous groups to improve cardiac metabolism, including a reduced incidence of arrhythmia and increased coronary blood flow (Hayward et al., 2015; Hulot et al., 2016). However, the benefits of administering SERCA2a were shown to be diminished in a myocardium with a limited energy supply (Pinz et al., 2011), suggesting increasing SERCA2 levels may only be beneficial in non-ischemic tissue. In humans, directly restoring SERCA2 levels has been achieved using gene transfer. Adeno-associated virus1.SERCA2a (AAV1.SERCA2a) gene transfer successfully restored the Ca^2+^ transient and contraction in isolated failing human cardiomyocytes (del Monte et al., 1999) and in animal models of HF, SERCA2a gene transfer was shown to increase survival and improve cardiac hemodynamics and function (del Monte et al., 2001, 2004; Kawase et al., 2008) (Figure 4 and Table 2). Given that SERCA2a gene transfer improved outcome in animal models of HF, AAV1.SERCA2a was administered in Phase I and Phase II clinical trials [ClincalTrials.gov identifiers NCT00534703 and NCT00454818] (Jessup et al., 2011; Zsebo et al., 2014; Greenberg et al., 2016). The results of the initial Phase I and IIa trials suggested that AAV1.SERCA2a treatment reduced the number of cardiac events in HF patients (Jaski et al., 2009; Jessup et al., 2011); however, larger Phase IIb clinical trials failed to show improvement in any primary or secondary endpoints, leading to their early termination (Hulot et al., 2017). The failure of these clinical trials suggests the dosage and delivery method of AAV1.SERCA2a may require optimizing (Hulot et al., 2016) [SERCA2a gene therapy in HF has been extensively reviewed elsewhere (Samuel et al., 2018)]. Despite the lack of promising results for AAV1.SERCA2a, clinical trials are underway for istaroxime, a dual functional luso-inotropic agent which both stimulates SERCA2a and inhibits Na^+^/K^+^ ATPase (Micheletti et al., 2002) (Figure 4 and Table 2). Istaroxime increases Ca^2+^ uptake into the SR by relieving PLN-mediated inhibition of SERCA2a through cAMP/PKA-depending mechanism (Ferrandi et al., 2013). In animal models of HF, intravenous istaroxime improved cardiac function without causing any arrhythmic events (Micheletti et al., 2007; Sabbah et al., 2007). Phase I-II clinical trials demonstrated the safety of istaroxime in HF patients and showed beneficial effects on cardiac contractility (Ghali et al., 2007). The follow-up Phase IIa clinical trial, ‘HORIZON-HF,’ showed a reduction in diastolic stiffness and increase in contractility in HFrEF patients receiving istaroxime when compared to placebo (Shah et al., 2009). Additional clinical trials for istaroxime are on-going [ClincalTrials.gov identifiers NCT02617446 and NCT02477449].

**TABLE 2:** Therapeutic targeting of Ca^2+^-channels and pumps in cardiac hypertrophy, HF and cardiac arrhythmia.

**Target**	**Pre-clinical**	**FDA approved**	**Clinical trial identifier**	**Status**	**Results**
NCX	Neucardin		NCT01251406	C	None posted
			NCT01541202	T	N/A
			NCT03388593	R	N/A
	JK07		NCT04210375	NYR	N/A
	KB-R7943		Pre-clinical	N/A	N/A
	SEA-0400		Pre-clinical	N/A	N/A
	SN-6		Pre-clinical	N/A	N/A
	ORM-10962		Pre-clinical	N/A	N/A
PMCA4	ATA		Pre-clinical	N/A	N/A
	Caloxin 1B1		Pre-clinical	N/A	N/A
PMCA1	Caloxin 1B3		Pre-clinical	N/A	N/A
RyR2		Dantrolene	jRCTs61180059	R	N/A
		Phenytoin	Pre-clinical for HF	N/A	N/A
	JTV519		NCT00626652	C	None posted
	S107		Pre-clinical	N/A	N/A
		Flecainide	NCT01117454	C	Used with β-blockers, no safety concerns (Kannankeril et al., 2017)
SERCA2a	Istaroxime		NCT02617446	U	N/A
			NCT02477449	U	N/A
	AAV1.SERCA2a		NCT00534703	T	Only 5 participants, trial terminated early
			NCT00454818	C	No safety concerns in 3-year follow-up (Zsebo et al., 2014)
	N106		Pre-clinical	N/A	N/A
	Ad.SERCA2a		Pre-clinical	N/A	N/A
TRPC3	Pyr3		Pre-clinical	N/A	N/A
TRPC3/C6	GSK2332255B GSK2833503A		Pre-clinical	N/A	N/A
TRPV4	GSK2798745		NCT02497937	C	1 (/11) incident of a serious adverse event in treatment group
			NCT02119260	C	Safe, well-tolerated (Goyal et al., 2019)
	HC-067047		Pre-clinical	N/A	N/A
TRPM4	9-phenanthrol		Pre-clinical	N/A	N/A
TTCC		Mibefradil*			Initially safe (Bursztyn et al., 1997; Kobrin et al., 1997) but later withdrawn due to interference with metabolism of other medicines
MCU	Ruthenium red		Pre-clinical	N/A	N/A
	Ru_360_		Pre-clinical	N/A	N/A

Each therapy listed in Figure 4 is listed as having either pre-clinical or FDA-approved status. Any clinical trial information available, either through publication or as results published, is also presented. Clinical trials accessed through clinicaltrials.gov have an NCT number whereas those obtained through the Japan Registry of Clinical Trials are denoted by a jRCTs number. * Mibefradil was withdrawn from the market following FDA approval. C, completed; N/A, not applicable; NYR, not yet recruiting; R, recruiting; T, terminated; U, unknown.

Focus for novel SERCA2 therapeutics has also extended to SERCA2 post-translational modifications and mediators of SERCA2 expression. Gene-transfer of SUMO1 and S100A1 which both promote SERCA2 activity were shown to improve cardiac function in rodent and porcine models of HF (Most et al., 2004; Kho et al., 2011; Pleger et al., 2011; Tilemann et al., 2013). Moreover, a small-molecule SUMO1 activator, termed N106, has recently been developed which, through activating the SUMO-activating enzyme, leads to the SUMOylation of SERCA2 (Kho et al., 2015). In addition, microRNA’s upregulated in human HF also provide a novel therapeutic target as they can be inhibited using anti-*miR*’s. One such microRNA, microRNA-25, is upregulated in human HF and application of anti-*miR-25* following TAC improved survival and function by restoring SERCA2 activity (Wahlquist et al., 2014). However, none of these SERCA2 modifying mechanisms have been investigated in the clinical setting. SERCA2a and its regulator PLN can be modified by nitroxyl (HNO/NO^–^), the single electron reduced form of nitric oxide (Paolocci et al., 2001; Farah et al., 2018). HNO readily converts negatively charged thiol groups of proteins, such as cysteine, to disulfide residues; a feature which is reversible (Fukuto, 2019; Pinilla-Vera et al., 2019). HNO modifies cysteine residues on SERCA2a and PLN (and RyR2) which enhances Ca^2+^ cycling through reduced PLN-mediated SERCA2a inhibition (Froehlich et al., 2008; Farah et al., 2018). The original HNO donor used in proof-of-principal experiments, Angeli’s salt, is not stable at room temperature, therefore, first- and second-generation HNO donors have been developed with more favorable drug-like properties. One such second-generation HNO derivative to have undergone clinical testing is BMS-986231 (Figure 3 and Table 1). To date, there have been eight completed different clinical trials utilizing BMS-986231. Results from a Phase I clinical trial showed that BMS-986231 was well tolerated but that nearly half of healthy volunteers receiving BMS-986231 reported headaches; however, the authors noted that owing to BMS-986231 vasodilatory effects, the presence of headaches was not unexpected (Cowart et al., 2019) [clinicaltrials.gov identifier NCT03016325]. In a separate placebo-controlled, double-blind Phase IIa study, 34 patients were randomly assigned to receive one infusion of BMS-986231 (Tita et al., 2017) [clinicaltrials.gov identifier NCT02157506]. The results from this Phase IIa clinical trial also suggest BMS-986231 is safe and in HFrEF patients, exhibits a favorable hemodynamic profile (Tita et al., 2017). The success of these early Phase I and II clinical trials have led to the development of three further clinical trials to determine the effect of BMS-986231 on (1) symptomatic hypotension in HFrEF patients, (2) cardiac systolic and diastolic function in HFrEF patients, and (3) salt and water-handling in patients with HFrEF and HFmrEF (EF < 45%) [clinicaltrials.gov identifier(s) NCT03016325, NCT03357731, and NCT03730961, respectively] (Felker et al., 2019). To date, these studies have completed recruitment and are on-going.

#### NCX

Depending on the membrane potential and intracellular Ca^2+^ and Na^+^ gradients, NCX1 can act in ‘forward’ (Ca^2+^ ion out, 3 Na^+^ ions in) or ‘reverse’ (Ca^2+^ ion in, 3 Na^+^ ions out) mode. In a failing cardiomyocyte, NCX1 has been shown to operate in reverse mode which results in Ca^2+^ entry and a repolarizing current (Mattiello et al., 1998). Furthermore, elevated intracellular Ca^2+^ seen after I/R injury is caused by NCX1 operating in ‘reverse’ mode (Piper et al., 1996; Inserte et al., 2002; Imahashi et al., 2005). NCX1 is upregulated in human and murine models of HF (Hasenfuss et al., 1999; Menick et al., 2007) and over-expression of NCX is sufficient to impair cardiomyocyte function *in vitro* (Schillinger et al., 2000). However, as the function of NCX is also affected by Na^+^, and intracellular Na^+^ is increased in both human HF and models of HF (Bers and Despa, 2006), Ca^2+^ extrusion through NCX could be impaired due to Na^+^-loading observed in end stage HF (Louch et al., 2010; Li et al., 2012). Interestingly, following TAC there is an increase in NCX expression but no change in SERCA2a; however, when TAC progresses into HF, there is a downregulation in SERCA2 expression (Wang et al., 2001). Therefore, in HF there is a change in the NCX/SERCA2 ratio which has been implicated in impaired cardiac contraction and conduction (Pogwizd et al., 2001).

Historically, digoxin has been used to inhibit NCX; however, there is no improvement in mortality of patients receiving digoxin (Campbell and MacDonald, 2003; Goldhaber and Hamilton, 2010). It was recently observed that an increase in NCX1 seen in an animal model of HF could be attenuated by neuroglin-1β (Wang et al., 2019) (Figure 4). Neuroglin-1β has been extensively explored as a therapeutic option in HF and expression of recombinant human neuroglin-1β (rhNRG-1/also known as Neucardin) has undergone successful clinical trials; however, its full mechanism of action has not yet been determined (Galindo et al., 2014). Neuroglin-1β is a growth factor which, along with its receptors ErbB2-4, is critical for the cardiac response to stress (Lemmens et al., 2004; Galindo et al., 2014; Wang et al., 2019). Following pressure-overload induced cardiac hypertrophy, the expression of ErbB2 and B4 are increased during the cardiac compensation phase and reduced following the transition into HF (Rohrbach et al., 1999). Similarly, in the left ventricle of human failing myocardium, the expression of ErbB2 and B4 is reduced (Rohrbach et al., 2005). Animal models of hypertrophic- and ischemia-induced HF have been used extensively to show the benefit of recombinant neuroglin-1β (Galindo et al., 2014). Treatment with neuroglin-1β for 7 days was sufficient to prevent ventricular remodeling following volume-overload induced cardiac hypertrophy (Wang et al., 2019). At the molecular level, neuroglin-1β partially prevented the decrease in SERCA2a, LTCC and increase in NCX1 associated with HF (Wang et al., 2019). Therefore, neuroglin-1β may confer its cardioprotective effects through regulation of Ca^2+^-handling protein expression. Given the promising effects of recombinant neuroglin-1β treatment in animal models of HF, Phase II and III clinical trials were approved to determine the safety and efficacy of daily infusions of Neucardin (Galindo et al., 2014). The first Phase II clinical trials successfully demonstrated the safety, tolerability and efficacy of rhNRG-1 in patients with chronic HF (Gao et al., 2010; Jabbour et al., 2011) [clinicaltrials.gov identifier NCT01251406 and NCT01541202]. Following on from these early Phase II clinical trials, large-scale, multi-centre Phase III clinical trials have begun recruiting patients to determine the effect of Neucardin on all-cause mortality after 1 year [clinicaltrials.gov NCT03388593]. Furthermore, a Phase I clinical trial has recently been accepted to test the safety, tolerability and immunogenicity of the drug JK07 in HFrEF patients; JK07 is a human immunoglobulin IG1 monoclonal antibody containing an active polypeptide fragment of neuroglin-1 [clinicaltrials.gov identifier NCT04210375] (Figure 4 and Table 2). Future studies should aim to determine whether neuroglin-1β mediates NCX1, SERCA2a and LTCC channel expression in other animal models of HF and whether the efficacious effects of Neucardin in clinical trials is partly due to altered Ca^2+^ dynamics.

#### RyR2

There is a general consensus that SR-located RyR2 contributes to Ca^2+^ leak during HF which can cause (1) cardiac arrhythmias, (2) reduced Ca^2+^ SR content, (3) systolic dysfunction, and (4) altered cardiac energetics (Bers, 2014). The function of RyR2, the major cardiac isoform of the RyR, is tightly regulated though changes to its phosphorylation status, Ca^2+^ activation and inactivation sites and post-translational modifications (Roe et al., 2015). RyR2 channel gating is regulated by accessory proteins whilst its tetrameric confirmation is stabilized by FK506-binding protein-12.6 (FKBP12.6). Disassociation of FKBP12.6 from RyR2 is observed in some models of HF and is achieved through PKA-mediated hyperphosphorylation of RyR2 at Ser2808 (Marx et al., 2000). One study has demonstrated that transgenic mice with an absent PKA phosphorylation site on RyR2 are protected against MI-induced HF (Wehrens et al., 2006); however, others have shown that CaMKII-mediated RyR2 phosphorylation leads to altered Ca^2+^ sparks as opposed to PKA-mediated phosphorylation (Li et al., 2002; Guo et al., 2006). Such is the importance of CaMKII-mediated phosphorylation of RyR2 that it has been shown to promote the transition from hypertrophy into HF (Ling et al., 2009; Respress et al., 2012).

Changes to Ca^2+^ transfer between the SR and mitochondria impedes mitochondrial function in cardiomyocytes (Fernandez-Sanz et al., 2014; Ruiz-Meana et al., 2019), potentially contributing to cardiomyocyte deterioration in HF. Recently, a novel mechanism of RyR2 hyperglycation was shown to increase Ca^2+^ leak from the SR in cardiomyocytes of aged mice (Ruiz-Meana et al., 2019). Aging alone results in advanced glycation of RyR2, leading to increased SR Ca^2+^ leak, mitochondrial Ca^2+^ overload and mitochondrial dysfunction, independently of cardiac disease (Ruiz-Meana et al., 2019). Importantly, hyperglycation of RyR2 and increased mitochondrial Ca^2+^ was prevalent in atrial appendages from aged patients (75 years or above) and absent in young patients (Ruiz-Meana et al., 2019). Preventing the glycation of RyR2 by inhibiting dicarbonyl intermediaries or stimulating their detoxification may provide a preventative measure to attenuate mitochondrial damage associated with aged, failing cardiomyocytes.

RyR2 can be inhibited by dantrolene sodium, a derivative of the hydantoin class of compounds (Maxwell et al., 2012). Application of dantrolene normalizes the diastolic Ca^2+^ leak seen in HF; however, owing to its hepatotoxic side-effect, dantrolene is not suitable for long-term use (Durham et al., 1984) (Figure 4 and Table 2). Despite this, the ‘SHO-IN’ clinical trial seeks to determine the safety and efficacy of dantrolene on patients with chronic HF [Japanese Registry of Clinical Trials identifier 61180059] (Kobayashi et al., 2020). Recent work has shown that phenytoin, a member of the same class of hydantoin compounds, shares the same RyR2 inhibitory effects as dantrolene (Ashna et al., 2020). *In vitro* application of phenytoin to RyR2 channels isolated from failing human hearts shows that phenytoin reversibly inhibits RyR2 which subsequently inhibits diastolic cytoplasmic, but not systolic, Ca^2+^ release (Ashna et al., 2020). Interestingly, phenytoin is currently used clinically as a Na^+^ channel blocker to treat epilepsy, therefore there is scope to determine the potential therapeutic use of phenytoin in HF (Figure 4). Designing novel therapeutic targets for RyR2 may, in future, involve RyR2 accessory proteins which modulate its function. TRIC-A and B are SR-located K^+^ channels known to participate in ECC (Yazawa et al., 2007; Yamazaki et al., 2009b; Yang et al., 2016); both TRIC-A and TRIC-B knockout mice exhibit Ca^2+^ overload in the SR (Yamazaki et al., 2009a; Zhao et al., 2010). TRIC-A, but not TRIC-B, physically associates with RyR2 to enhance RyR2-dependent Ca^2+^ efflux from the SR; however, there is still a limited understanding of the function of TRIC-A-dependent RyR2 modulation under pathological conditions (Zhou et al., 2020).

#### TRP Channels

When compared to a healthy myocardium, TRPC1, C5, M4, and M7 are upregulated and TRPC4 and V2 are downregulated in the failing human heart (Dragun et al., 2019), suggesting each TRP channel may differentially contribute to CVD [reviewed extensively elsewhere (Falcon et al., 2019; Hof et al., 2019)]. Interestingly, in neonatal rat cardiomyocytes, stimulation with hypertrophy-inducing agents leads to the upregulation and subsequent activation of TRPC1 (Ohba et al., 2007) and C5 (Sunggip et al., 2018). Furthermore, in mouse models, global deletion of *Trpc1* attenuates pressure-overload induced hypertrophy through reduced activation of the calcineurin/NFAT pathway (Ohba et al., 2007; Seth et al., 2009). In addition to TRPC1 and C5, TRPC3 (Brenner and Dolmetsch, 2007) and C7 (Satoh et al., 2007) are also upregulated in hypertrophy-stimulated cardiomyocytes and TRPC6 overexpression induces cardiac hypertrophy in mice (Kuwahara et al., 2006). TRPC3 and C6 have been successfully co-targeted with the thiazole inhibitors GSK2332255B (GSK255B) and GSK2833503A (GSK503A), respectively (Washburn et al., 2013) (Figure 4 and Table 2). When these inhibitors were co-administered following pressure-overload induced hypertrophy in mice, both cardiomyocyte hypertrophy and cardiac fibrosis were reduced (Seo et al., 2014). Mechanistically, blockade of TRPC3 reduces phosphorylation of Ca^2+^-handling proteins in mouse embryonic stem cell-derived cardiomyocytes (Qi et al., 2016), which may alter downstream Ca^2+^-mediated signaling. More specifically, TRPC3 can be exclusively inhibited by Pyr-3 (Kiyonaka et al., 2009) (Figure 4 and Table 2). Pyr-3 inhibition of TRPC3 attenuates NFAT-mediated transcription and can prevent hypertrophy *in vitro* in ANG-II stimulated neonatal rat cardiomyocytes and *in vivo* following pressure overload-induced hypertrophy in mice (Kiyonaka et al., 2009). Other studies have also shown TRPC1 and TRPC3-C6 upregulation in failing human hearts when compared to non-failing myocardium (Bush et al., 2006; Morine et al., 2016). Interestingly, the same TRPC channels are also upregulated in the border zone of rats hearts after left anterior descending coronary artery ligation-induced MI (Dominguez-Rodriguez et al., 2018). Therefore, it appears that the TRPC family of channels contribute to the cardiac HF phenotype; however, more research is required in order to determine the functional effect of each upregulated TRPC channel in order to progress pre-clinical studies.

The TRPV family have also been extensively investigated; however, as mentioned above, there is no detectable upregulation of TRPV family members in the failing human myocardium (Dragun et al., 2019). Despite this, there is promising data to suggest blockade of TRPV4 may prove beneficial in HF patients; the pulmonary endothelial-localized TRPV4 channel has been shown to play a role in pulmonary venous pressure-induced formation of pulmonary oedema in HF (Thorneloe et al., 2012). In support of this, TRPV4 is upregulated in human lungs derived from patients with congestive HF (Thorneloe et al., 2012). As such, TRPV4 channel blockade with the first in class orally active pharmaceutic GSK2798745 successfully prevents the formation of and reverses established pulmonary oedema in acute and chronic HF models (Thorneloe et al., 2012) (Figure 4 and Table 2). The positive data obtained from TRPV4 inhibition in animal models led to a Phase I clinical trial to investigate the safety and efficacy of inhibiting TRPV4 with GSK2798745 (Goyal et al., 2019). Results of this first in man trial has shown there are no adverse effects or safety issues in both healthy volunteers and patients with mild to moderate HF (Goyal et al., 2019) [clinicaltrials.gov identifiers NCT02497937 and NCT02119260]. In addition, TRPV4 is activated in the murine model of I/R injury (Dong et al., 2017). Mechanistically, activation of TRPV4 following I/R leads to increased Ca^2+^ influx and generation of ROS (Wu et al., 2017). Therefore, chemical inhibition of TRPV4 with the selective antagonist HC-067047 prevents aberrant TRPV4 activation which leads to a reduction in infarct size and improved cardiac function following I/R injury (Dong et al., 2017). Furthermore, both TRPV1 and V2 contribute to the inflammatory response following MI; however, mice harboring a global deletion of TRPV1 show an exacerbated inflammatory response 3- and 7-days post-MI whereas mice with a global knockout of TRPV2 show improved cardiac recovery following MI due to a reduction in peri-infarct macrophages (Huang et al., 2009; Entin-Meer et al., 2017; Falcon et al., 2019).

Some of the TRPM and TRPP channels exhibit the highest levels of expression in basal ventricular myocytes, amongst these are TRPM7, M1 and M4 and TRPP1 and P2 (Chevalier et al., 2018). As mentioned, both TRPM4 and M7 levels are elevated in the failing human ventricle myocardium (Dragun et al., 2019); however, TRPM7 mRNA was shown to be reduced in the left ventricle of patients with ischemic cardiomyopathy (Ortega et al., 2016), therefore the underlying cause of HF may influence the expression pattern of TRP channels. TRPM7 is expressed in the SAN and has a known role in modulation of cardiac automaticity (Sah et al., 2013a,b); however, TRMP7 also promotes cardiomyocyte proliferation during early cardiogenesis (Sah et al., 2013a,b). TRMP7 kinase dead mice exhibit significant cardiac hypertrophy and upregulation of pro-inflammatory mediators, driven by a Mg^2+^- but not a Ca^2+^-sensitive mechanism (Rios et al., 2020). Therefore, TRPM4 may provide a better option to therapeutically target Ca^2+^ mishandling. Interestingly, despite the upregulation of TRPM4 in failing human hearts, the channel itself is impermeable to Ca^2+^; however, it is both Ca^2+^- and voltage-activated (Nilius et al., 2003, 2005; Wang et al., 2018). TRMP4 expression is upregulated by 50-fold in spontaneously hypertensive rats when compared to controls (Guinamard et al., 2006). Furthermore, global deletion of *Trpm4* causes eccentric cardiac hypertrophy with aging which is caused by neonatal cardiomyocyte hyperplasia (Demion et al., 2014). In a cardiomyocyte-specific *Trpm4* knockout mouse (*Trpm*^cko^), induction of cardiac hypertrophy with ANG-II resulted in increased hypertrophy in *Trpm4*^cko^ mice when compared to controls (Kecskes et al., 2015). TRPM4 activity can be exclusively inhibited by 9-phenanthrol (Guinamard et al., 2014). Most studies using 9-phenanthrol have aimed to determine the contribution of TRMP4 to smooth muscle cell contraction (Guinamard et al., 2014); however, application of 9-phenanthrol to isolated rat hearts in a model of I/R injury reduces infarct size and improves contractility (Wang J. et al., 2013).

#### PMCAs

The PMCAs are rapidly emerging as potential signaling mediators (Armesilla et al., 2004; Oceandy et al., 2007; Cartwright et al., 2011; Stafford et al., 2017); however, to date, only a role for PMCA4 has been elucidated in a mouse model of TAC-induced pressure-overload hypertrophy. In the study by Mohamed et al. (2016), global knockout of *Atp2b4* was shown to attenuate the pressure-overload response; however, the anti-hypertrophic effects of PMCA4 knockout could only be replicated in fibroblast-specific and not in cardiomyocyte-specific knockout mouse models (Mohamed et al., 2016; Stafford et al., 2017). Non-myocyte cells such as cardiac fibroblasts and endothelial cells account for around 70% of total cardiac cells (Pinto et al., 2016), and are able to secrete factors which can influence adjacent cardiomyocyte signaling. Interestingly, fibroblast-specific deletion of PMCA4 leads to significant upregulation of secreted frizzled related protein 2 which is thought to confer cardioprotective properties by inhibiting the Wnt pathway in neighboring cells (Mohamed et al., 2016). Despite this role for PMCA4 in cardiac hypertrophy, there is no evidence that *human* ATP2B4 is associated with CVD; genome-wide association studies (GWAS) have to date been unable to associate SNPs within ATP2B4 with any CVD (Tabara et al., 2010).

In contrast to GWAS data for *ATP2B4*, SNPs located in *ATP2B1*, the gene encoding PMCA1, have been strongly associated with CVDs, including hypertension, coronary artery disease and MI (Tabara et al., 2010; Takeuchi et al., 2010; Wang Y. et al., 2013; Xu et al., 2016; Jamshidi et al., 2018; Sombie et al., 2019). The association of *ATP2B1* with hypertension has been shown in numerous studies across different ethnic groups; to date, there are at least six different SNPs within *ATP2B1* which are associated with elevated blood pressure (Tabara et al., 2010; Takeuchi et al., 2010; Wang Y. et al., 2013; Xu et al., 2016; Jamshidi et al., 2018; Sombie et al., 2019). In mice, vascular smooth muscle cell-specific PMCA1 knockout mice exhibit elevated blood pressure (Kobayashi et al., 2012). Mechanistically, loss of PMCA1 from vascular smooth muscle cells leads to enhanced expression of LTCC and increased Ca^2+^ concentration which may affect smooth muscle cell stiffness and subsequently, vascular tone (Okuyama et al., 2018; Zhu et al., 2019). Furthermore, PMCA1 global heterozygous mice have increased hypertension with aging, which is preceded by vascular remodeling (Little et al., 2017). Taken together, PMCA1 appears to play a functional role in the development of hypertension through modulation of Ca^2+^. Whilst more research is required to determine the contribution of PMCA1 to hypertrophy and HF, there is scope to therapeutically target PMCA1 as a modulator of hypertension to prevent CVD (Little et al., 2016).

The PMCAs are typical ATPases and therefore present a suitable novel therapeutic target (Figure 4 and Table 2). ATPases have been clinically targeted to treat hypotension and some cardiac arrhythmias; ouabain inhibits Na^+^/K^+^-ATPase and omeprazole inhibits H^+^/K^+^-ATPase. Therapeutically, the two main cardiac isoforms of PMCA have been successfully targeted *in vitro*; the caloxins, a class of PMCA inhibitors, show differential specificity for each PMCA isoform (Pande et al., 2011). Caloxin 1B3 shows increased specificity for PMCA1 over PMCA4 and results in increased Ca^2+^ in endothelial cells (Szewczyk et al., 2010; Pande et al., 2011), whereas caloxin 1B1 is predominantly PMCA4-isoform specific (Pande et al., 2006). PMCA4 has also been successfully targeted with aurintricarboxylic acid (Mohamed et al., 2013a). Application of aurintricarboxylic acid *in vivo* was able to prevent and also reverse hypertrophy in the murine TAC model of pressure overload (Mohamed et al., 2013b), suggesting inhibition of PMCA4 may provide an interesting therapeutic target.

#### MCU

Ca^2+^ is also required for effective mitochondrial function. Mitochondrial Ca^2+^ cycling is achieved through the inner mitochondrial membrane located MCU (Figure 2). Mitochondria are abundant in cardiomyocytes owing to their function in the synthesis of ATP (Kwong, 2017). In the process of ATP generation, mitochondria utilize Ca^2+^ to activate pyruvate dehydrogenase, ATP synthase and other enzymes involved in the redox reaction, as such [Ca^2+^] in the inner matrix is tightly regulated by MCU (Kwong, 2017). MCU levels are elevated in the failing human heart and in animal models of HF (Zaglia et al., 2017; Yu et al., 2018). Under pathological conditions, overload of mitochondrial Ca^2+^ is associated with reduced cell viability, as well as apoptotic and autophagic cell death (Pinton et al., 2008). Interestingly, recent work suggests MCU is dispensable for normal cardiac function; cardiomyocyte-specific *Mcu*^–/–^ mice show no overt phenotype under basal conditions, suggesting MCU is not required for maintaining cardiac homeostasis (Luongo et al., 2015). Furthermore, *Mcu*^–/–^ mice do not experience mitochondrial Ca^2+^ overload following I/R injury which protects them against cell death and adverse cardiac remodeling (Luongo et al., 2015). Similarly, following TAC-induced pathological cardiac hypertrophy, mice treated with the MCU chemical inhibitor RR show improved survival, cardiac function and maintained mitochondrial integrity (Yu et al., 2018) (Figure 4 and Table 2). Treatment of isoprenaline stressed H9C2 cells with RR *in vitro* confirmed its ability to increase both autophagy and mitophagy (Yu et al., 2018). Taken together, inhibition or depletion of MCU appears beneficial for attenuating the pathological response to ischemic and hypertrophy stimuli. However, the use of RR has been shown to interfere with Ca^2+^ flux through mitochondrial-located RyR1 and membrane bound LTCC (Beutner et al., 2001). Therefore, developing more specific inhibitors of MCU to combat HF offers an alternative to targeting the ‘typical’ Ca^2+^-handling proteins.

## Cardiac Arrhythmias

Cardiac arrhythmias are a class of conditions describing abnormalities in the heart’s normal rhythm. Arrhythmias are a leading cause of morbidity and mortality, and can lead to stroke, deterioration of ventricular function, and sudden cardiac death (Kass and Clancy, 2006). Arrhythmic events can vary in terms of speed, rhythm, duration and location in the heart, and can present in numerous ways. The main types of arrhythmia include bradycardias (slow but regular heart rhythms) and tachycardias (faster than normal heart rates), including supraventricular tachycardias (tachycardia originating in the atria). Other common arrhythmias are AF (an irregular, often fast heart rhythm) and heart block (the heart beats slower than normal due to a delay in conduction) (Keating and Sanguinetti, 2001; Kass and Clancy, 2006).

Arrhythmogenesis is complex and multifactorial. Numerous genetic mutations have been shown underlie primary arrhythmia disorders, such as LQTS and CPVT. Within these conditions, the underlying genetic defect determines the arrhythmia phenotype, including the physiological triggers which incite arrhythmic events in these patients (Wilde and Bezzina, 2005). In addition, pathological and physiological factors can influence cardiac electrical remodeling, and therefore trigger arrhythmia development, including HF, hypertension, and coronary heart disease. For example, HF can result in structural and hemodynamic dysfunction, electrical remodeling (including those impacting Ca^2+^ handling), and metabolic changes which pre-dispose the development of arrhythmias (Masarone et al., 2017). Furthermore, susceptibility to these acquired arrhythmic disorders can be influenced by genetic factors (Wilde and Bezzina, 2005).

### Calcium Handling Proteins in Arrhythmia Development and Their Potential as Novel Therapeutic Targets

Calcium handling in the myocardium is key to the generation and control of the heartbeat. Intracellular Ca^2+^ remodeling has been identified as a major factor in the development of arrhythmias and current approved therapies for arrhythmic disorders include Ca^2+^ channel blockers (Deo et al., 2017). Advancements in human genetic studies have highlighted mutations in Ca^2+^ handling proteins, as well as Ca^2+^ channels, that can result in the development of arrhythmias. In addition, remodeling of Ca^2+^ handling proteins has been identified in patients with acquired arrhythmic conditions (Landstrom et al., 2017). Therefore, Ca^2+^ handling proteins are promising therapeutic targets for the treatment of arrhythmic disorders. In the following section, we will discuss the current research aimed at targeting Ca^2+^ handling proteins in arrhythmia development.

### The Role of Ca^2+^ in Arrhythmia Development

The mechanisms underlying arrhythmia development are generally divided into three categories: automaticity, triggered activity, and re-entry (Antzelevitch and Burashnikov, 2011). The role of Ca^2+^ in the generation of arrhythmias has been previously extensively reviewed (see Antzelevitch and Burashnikov, 2011; Kistamas et al., 2020). Automaticity is the ability of a cell to spontaneously depolarize and generate an AP. Under normal conditions, automaticity is restricted to the cardiac conduction system, specifically the SAN pacemaker cells which initiate cardiac contraction (Antzelevitch and Burashnikov, 2011). Abnormal automaticity occurs when the automaticity of the SAN is altered or when other cells begin to fire spontaneously. Spontaneous beating of the SAN is regulated by a coupled system of the surface membrane clock (inactivation and deactivation of cardiac ion channels) and the intracellular Ca^2+^ clock (rhythmic SR Ca^2+^ release) (Joung et al., 2011). Unsurprisingly, malfunction of both clocks has been associated with arrhythmia development. In relation to Ca^2+^ handling, Ca^2+^ clock remodeling (unresponsiveness to β-adrenergic stimulation and downregulation of RyR2) has been identified in SAN dysfunction (Joung et al., 2011). In addition, triggered oxidation and activation of CaMKII has been shown to result in SAN cell death which may contribute to SAN dysfunction (Huke and Knollmann, 2011).

Triggered activity refers to voltage oscillations termed afterdepolarizations which are often Ca^2+^-mediated. Early afterdepolarizations (EADs) occur during the plateau phase of the cardiac AP and are associated with LQTS and ventricular tachyarrhythmias (associated with HF) (Landstrom et al., 2017). They are triggered by a reduction in repolarization reserve as a result of an increase in an inward current or a decrease in an outward current (Huang et al., 2018). A main contributor to EAD initiation is an increase in L-type Ca^2+^ current (*I*_CaL_) which drives the EAD upstroke. This can occur due to a reactivation of the current during the repolarization phase caused by a reduction in the outward IK current or by CaMKII-mediated phosphorylation of *I*_CaL_ (Weiss et al., 2010; Landstrom et al., 2017). In addition, increased NCX current (*I*_NCX_) can contribute to EAD. Spontaneous Ca^2+^ release events either prior to the completion of repolarization or during the initial phase of EADs can cause an increase in *I*_NCX_ activity which may delay repolarization and allow *I*_CaL_ to reactivate (Zhong et al., 2018).

Delayed afterdepolarizations (DADs) typically occur after repolarization and are caused by Ca^2+^ overload driving spontaneous Ca^2+^ release from the SR (Fink et al., 2011). Ca^2+^ release from the SR activates NCX (Ca^2+^-sensitive Cl^–^- and non-specific ion currents can also be activated) leading to depolarization of the membrane. If the excitation threshold is reached, *I*_Na_ becomes activated and an AP is triggered (Fink et al., 2011). An increase in Ca^2+^ influx or a decrease in Ca^2+^ efflux can lead to an increase in SR Ca^2+^ content and drive the Ca^2+^ waves that activate NCX. The SR threshold for Ca^2+^ waves is thought to be dependent on the properties of RyR, with the opening of RyR decreasing the threshold for Ca^2+^ release (Venetucci et al., 2008). Elevated Ca^2+^ levels increase the open probability of RyR, making them more sensitive to stochastic Ca^2+^ release, ultimately leading to Ca^2+^ release (Fink et al., 2011). Phosphorylation of RyR at the PKA site or the CaMKII site can also increase RyR calcium sensitivity and open probability (Wehrens et al., 2004a). Modulation of RyR has been attributed to arrhythmias associated with HF and CPVT (caused by an underlying RyR mutation) (Venetucci et al., 2008).

Re-entry underlies the formation of arrhythmias due to abnormal impulse conduction. Here an electrical impulse fails to terminate and instead re-excites the myocardium. For re-entry to occur there must be an abnormal electrical circuit, slow conduction and a unidirectional block (Antzelevitch and Burashnikov, 2011). Changes to Ca^2+^ handling can cause cardiac tissue to become vulnerable to re-entry arrhythmias by increasing the dispersion of refractoriness, leading to unidirectional conduction block, and slowing of conduction (Weiss et al., 2011). Subthreshold EADs and DADs cause electrical homogeneity by increasing dispersion of refractoriness and dispersion of excitability, respectively (Landstrom et al., 2017; Kistamas et al., 2020). In addition, cardiac alternans can act as a substrate from the initiation of re-entry and can arise due to Ca^2+^ cycling dysfunction. Cardiac alternans present as beat to beat variations in Ca^2+^ transient amplitude (Ca^2+^ alternans), AP duration (APD alternans), or contraction amplitude (mechanical alternans) (Kistamas et al., 2020). Changes in the level of SR Ca^2+^ content, reduced SERCA activity, and changes in the refractoriness have been proposed as mechanisms for the generation of Ca^2+^-derived alternans. DADs can also result in Ca^2+^ alternans and subsequent APD alternans (Kistamas et al., 2020).

### Ca^2+^-Handling Proteins in Arrhythmia Development; Ca^2+^ Channels, Pumps and Exchangers

#### RyR2

Abnormal RyR2-mediated Ca^2+^ release from the SR can lead to both atrial and ventricular arrhythmias, some primary arrhythmia disorders including CPVT and forms of acquired arrhythmias associated with heart disease (AF during HF) (McCauley and Wehrens, 2011). To diminish arrhythmic events, therapies have been developed to reduce and stabilize abnormal RyR activity, in order to inhibit diastolic SR Ca^2+^ release whilst maintaining the normal SR Ca^2+^ release during systole.

Dantrolene sodium is an approved muscle relaxant which is used for the treatment of malignant hyperthermia, caused by mutations in the skeletal RyR (RyR1) (McCauley and Wehrens, 2011). Dantrolene binds to the N-terminal of RyR1 to stabilize the inter-domain interactions required for the closed state of the RyR Ca^2+^ channel (Kobayashi et al., 2005). Further studies have shown dantrolene binds to RyR2 in a similar manner to improve the function of the receptor, suggesting the use of dantrolene in the treatment of arrhythmias (Figure 4) (Kobayashi et al., 2009). In a study using a knock-in mouse model carrying a human CPVT-RyR2 mutation (R2475S), dantrolene inhibited CPVT-related tachycardia (Kobayashi et al., 2010). Furthermore, in a cohort of CPVT-1 patients, dantrolene had an anti-arrhythmic effect, and reduced the number of premature ventricular complexes. This anti-arrhythmic response was also evident in induced pluripotent stem cell derived CPVT1 cardiomyocytes (Penttinen et al., 2015). In addition to CPVT, dantrolene has been shown to prevent the occurrence of DADs in failing cardiomyocytes isolated from a canine model (Kobayashi et al., 2009) and have a protective effect against arrhythmic events in animal models of I/R (Balam Ortiz et al., 1999; Boys et al., 2010). Together the data suggest dantrolene is a promising therapy to attenuate Ca^2+^-mediated arrhythmias associated with HF. An early Phase I clinical trial aimed to assess the feasibility of intravenous delivery of dantrolene to reduce ventricular arrhythmias in patients with structural heart disease [clinicaltrials.gov identifier NCT04134845]. While no results have yet been posted, the study highlights the potential of RyR2 inhibition for the treatment of arrhythmias.

JTV519 (also known as K201) is a 1,4-Benzothiazepines derivative initially identified to suppress sudden cardiac cell death associated with Ca^2+^ overload (Kaneko et al., 2009). Further studies identified RyR2 as a target of JTV519, with JTV519 having a protective effect in models of AF as a result of the restoration of RyR function (Figure 4 and Table 2) (Kumagai et al., 2003; Vest et al., 2005). PKA hyperphosphorylation of RyR2 and a reduction in the RyR2 channel-stabilizing subunit calstabin2 has been identified in AF. In a canine model of AF, treatment with JTV519 restored calstabin2 levels, stabilizing the closed state of RyR2 and reducing Ca^2+^ leak (Vest et al., 2005). A Phase 2 clinical trial evaluated the effects of JTV519 on sinus rhythm restoration in patients with AF [clinicaltrials.gov identifier NCT00626652], although no data is available. In terms of ventricular arrhythmias, a study using a calstabin2 global heterozygous knockout found pre-treatment with JTV519 prevented exercise-induced ventricular arrhythmias and sudden cardiac death associated with depletion of calstabin2. In the same study, JTV519 had no effect on calstabin2 knockout mice highlighting the requirement of calstabin2 for the antiarrhythmic effect of JTV519 (Wehrens et al., 2004b). Another 1,4-Benzothiazepines derivative is S107 which, like JTV519, can enhance RyR2 and calstabin2 binding to stabilize RyR2. S107 has been shown to be more specific than JTV519, with the latter known to interact with other cardiac ion channels including LTCC (Andersson and Marks, 2010). In a mouse model, S107 increased the binding of calstabin2 to RyRs carrying a CPVT mutation (R2474S) and inhibited associated arrhythmias (Figure 4) (Lehnart et al., 2008). The antiarrhythmic effect of S107 was further identified using a human induced pluripotent stem cell model of CPVT (Sasaki et al., 2016).

Flecainide is a trifluoroethoxybenzamide approved for the treatment of AF and/or supraventricular tachycardia. Importantly, flecainide is not recommended to patients with underling structural heart disease as the drug can be pro-arrhythmic in this environment (Andrikopoulos et al., 2015). While flecainide is classed as a type IC antiarrhythmic drug (a Na^+^ channel blocker) it is worth noting here the drugs ability to inhibit RyR2 action by reducing the duration of channel opening (Figure 4). Through this role as an RyR inhibitor, flecainide has been shown, in a mouse model of CPVT, to prevent ventricular tachycardia by suppressing spontaneous Ca^2+^ release. In addition, flecainide prevented exercise-induced ventricular arrhythmias in two patients carrying a CPVT-linked RyR mutation who were refectory to conventional therapy (Watanabe et al., 2009).

#### NCX

NCX has been identified to have a dual role in arrhythmogenesis. When Na^+^ levels are high, NCX acts in the reverse model to bring Ca^2+^ into the cell which can result in Ca^2+^ overload. When acting in the forward mode, the inward current formed during Ca^2+^ release can result in DADs (Antoons and Sipido, 2008). Increased NCX activity has been shown to underlie arrhythmogenesis in numerous conditions including HF and AF, making NCX inhibition an attractive arrhythmia treatment.

First generation NCX blockers such as aprinidine and bepridil displayed anti-arrhythmic effects. However, these NCX blockers lacked selectivity and were suggested to only have modest NCX blocking effects at therapeutic concentrations (Hobai and O’Rourke, 2004). KB-R7943 was the first NCX blocker with reported mode-selectivity, shown to be a more specific blocker of NCX in the reverse mode (Elias et al., 2001). Animal studies have shown KB-R7943 to be protective against Ca^2+^ overload and able to suppress reoxygenation-induced arrhythmias (Figure 4) (Ladilov et al., 1999; Mukai et al., 2000). In addition, KB-R7943 suppressed afterdepolarizations and ventricular tachyarrhythmias in a rabbit MI mode l (Chang et al., 2015). Paradoxically, this study also found KB-R7943 to enhance the inducibility of arrhythmias in this model (Chang et al., 2015), with another study showing KB-R7943 had no effect on I/R arrhythmias (Miyamoto et al., 2002). Further studies have also shown KB-R7943 to inhibit the LTCC current and various K^+^ currents, which brings into question the use of the drug in the treatment of arrhythmias (Tanaka et al., 2002).

SEA-0400 has been described as the most potent and selective NCX inhibitor compared to KB-R7943 (Tanaka et al., 2002). Like KB-R7943, SEA-0400 has been suggested to have a higher potency for the forward NCX mode (Lee et al., 2004) and has been shown to have a cardioprotective and antiarrhythmic effect, although results vary in different models (Figure 4). In a rat model of myocardial I/R injury, SEA-0400 improved cardiac function, and reduced the incidence of ventricular fibrillation and mortality (Takahashi et al., 2003). In contrast, however, the drug had no effect on the incidence of ventricular tachycardia and fibrillation in a larger animal model of I/R injury (Nagasawa et al., 2005). This study also investigated arrhythmias induced by digitalis. Here, SEA-0400 reduced the incidence of induced tachyarrhythmias although 2/8 animals experienced AV block and cardiac standstill (Nagasawa et al., 2005). This differential arrhythmic effect may be dependent on the expression levels of NCX. A study found SEA-0400 to be antiarrhythmic in mouse models with increased NCX expression, such as HF and AF, but in conditions with reduced NCX expression, SEA-0400 was found to increase Ca^2+^ transient amplitude and spontaneous Ca^2+^ transients, ultimately acting proarrhythmic (Bogeholz et al., 2017). These findings suggest that in clinical conditions in which NCX function is reduced, such as diabetic cardiomyopathy, further NCX inhibition could trigger arrhythmias by reducing NCX function below the level required for balanced Ca^2+^ dynamics (Bogeholz et al., 2017).

The antiarrhythmic benefits of blocking NCX with KB-R7943 and SEA-0700 has driven further studies to identify novel NCX inhibitors including SN-6 and ORM-10962 (Figure 4 and Table 2). While SN-6 has been shown to have cardioprotective effects, the potential of the benzyloxyphenyl derivative in treating HF-associated arrhythmias is hindered by its ability to also increase diastolic Ca^2+^ and block *I*_Ca_ (Gandhi et al., 2013). ORM-10962 is a potent NCX blocker which can inhibit both forward and reverse NCX currents and which has no significant effect on other cardiac ion currents. The drug has been shown to attenuate digoxin-induced DAD in isolated ventricular Purkinje fibers but had no effect on arrhythmias related to I/R injury (Kohajda et al., 2016). The contrasting effects of NCX blockers on arrhythmogenesis under different conditions highlights the need for further studies to explore the use of NCX blockers.

Interestingly, another therapeutic approach has been to stimulate the forward mode of NCX to reduce cytosolic Ca^2+^ and attenuate Ca^2+^ overload. Heparin oligosaccharides have been shown to directly interact with NCX and accelerate its activity (de Godoy et al., 2018). In a model of I/R, trisulfated heparin disaccharide and low molecular weight heparins had a significant antiarrhythmic effect, as a result of increased NCX forward activity. While it is worth nothing that both agents are known to target LTCC and Na^+^ channels, the mechanism of NCX acceleration appears to be a promising antiarrhythmic target (de Godoy et al., 2018).

#### SERCA

SERCA and its inhibitory protein PLN have been identified as important regulators of cardiac contractility and therefore potential antiarrhythmic targets (Antoons and Sipido, 2008). Impaired SERCA activity in HF results in dysregulation of systolic and diastolic function which can lead to cardiac arrhythmias. Increasing SERCA activity is a way of improving cardiac function in HF. In terms of arrhythmogenesis an increase in SERCA activity could theoretically result in Ca^2+^ overload and ultimately be arrhythmogenic (Antoons and Sipido, 2008). Encouragingly however, studies focusing on HF have found an increase in SERCA2 expression to be antiarrhythmic. In pre-clinical models of HF, increased SERCA2 expression through adenoviral delivery (Ad.SERCA2a) improved intracellular Ca^2+^ handling, restored contractility and attenuated ventricular arrhythmias (Figure 4) (del Monte et al., 2004; Lyon et al., 2011; Cutler et al., 2012). Other direct therapies to activate SERCA2 or inhibit PLN binding have been studied. Istaroxime, a Na^+^/K^+^ ATPase inhibitor which also stimulates SERCA, has been shown to have a moderate antiarrhythmic effect (Figure 4) (Bossu et al., 2018). Several clinical trials have assessed istaroxime in HF, highlighting the promise of the drug in cardiac disorders. However, it is worth noting is not known if higher doses of istaroxime could stimulate further arrhythmic episodes through Na^+^/K^+^ ATPase inhibition (Bossu et al., 2018).

#### T-Type Ca^2+^ Channel

As previously described, LTCC blockers are an approved treatment strategy for some arrhythmic conditions. The T-type Ca^2+^ channel has been also investigated as an antiarrhythmic target. T-type Ca^2+^ channels are localized in the pacemaker regions of the heart (the SAN, AVN and Purkinje fibers) where they are thought to contribute to SAN pacemaker activity and atrioventricular conduction (Mangoni et al., 2006). Inactivation of T-type Ca^2+^ channels results in bradycardia and delayed atrioventricular conduction, highlighting the importance of the channel in cardiac rhythm (Mangoni et al., 2006). Several studies have investigated the effect of blocking T-type Ca^2+^ channels with mibefradil, a tetralol derivative which inhibits T-type Ca^2+^ channels but is also known to block *I*_CaL_. Treatment with mibefradil results in a dose-dependent decrease in heart rate (Brogden and Markham, 1997), and prevention of ischemic-induced ventricular fibrillation (Figure 4) (Billman and Hamlin, 1996). However, the drug has been reported to result in toxicity, inducing TdP due to QT_c_ prolongation (Glaser et al., 2001).

#### CaMKII

CaMKII dysfunction is a common hallmark of many cardiac pathologies including HF. CaMKII-mediated arrhythmias have been identified in HF, AF, reperfusion injury, and genetic arrhythmic conditions including LQTS. Changes in CaMKII expression can influence the function of key cardiac ion channels and result in arrhythmogenesis (Mustroph et al., 2017). CaMKII-mediated hyperphosphorylation of RyR2 leads to spontaneous Ca^2+^ release events which can be proarrhythmic in disease conditions associated with Ca^2+^ leak. CaMKII can also drive Ca^2+^ overload through phosphorylation of Na_v_1.5 and subsequent reduction in NCX-mediated Ca^2+^ extrusion. In addition, an increase in CaMKII activity can result in prolonged repolarization through an increase in *I*_CaL_, an increase in Na^+^ influx, and reduced *I*_K_ function (Mustroph et al., 2017). This makes the inhibition of CaMKII is a promising antiarrhythmic approach.

KN-93, a CaMKII inhibitor, which works by binding directly to Ca^2+^/CaM, disrupts the ability of Ca^2+^/CaM to interact with CaMKII and activate the kinase (Wong et al., 2019). Inhibition of CaMKII by KN-93 has been shown to be antiarrhythmic in several models (Figure 3). In a RyR2^R4496C±^ knock-in mouse model of CPVT, KN-93 prevented catecholamine-induced sustained ventricular tachyarrhythmias (Liu et al., 2011). Further studies using induced pluripotent stem cells generated from a CPVT patient carrying a RyR2 mutation, showed KN-93 to drastically reduce DADs induced by catecholaminergic stress as well as stabilize Ca^2+^ activation under β-adrenergic stimulation to a pattern seen in normal tissue (Di Pasquale et al., 2013). CaMKII inhibition by KN-93 was also shown to reduce the incidence of stress-induced ventricular tachycardia in a junction knock-out mouse model which presents with a phenotype similar to CPVT (RyR2 channels display CaMKII-dependent hyperphosphorylation). Here, KN-93 also reduced the number of spontaneous Ca^2+^ aftertransients and aftercontractions under stress conditions (Tzimas et al., 2015). It is worth noting that in relation to CPVT, AAV-mediated delivery of a CaMKII peptide inhibitor (autocamtide-2-related inhibitory peptide, AIP) has been shown to suppress ventricular arrhythmias induced by either β-adrenergic stimulation or programmed ventricular pacing. AAV-GFP-AIP delivery also suppressed abnormal Ca^2+^ release events in induced pluripotent stem cells derived from two patients with two distinct CPVT pathogenic mutations (Bezzerides et al., 2019). The advantage of using a gene therapy approach over classic CaMKII blockers, including KN-93, is that it allows for cardiomyocyte-specific CaMKII inhibition with limited extracardiac effects.

In addition to CPVT, KN-93 has been shown to be effective in several other models of cardiac arrhythmias. Increased CaMKII-dependent SR Ca^2+^ leak is a hallmark of AF. Diastolic SR Ca^2+^ release via leaky RyR2 is hypothesized to contribute to arrhythmogenesis. In Ryr^2R176Q/+^ mice (exhibiting a gain-of-function defect in RyR), CaMKII inhibition prevented arrhythmias induced by rapid atrial pacing (Chelu et al., 2009). Studies have also indicated that KN-93 is effective at reversing the diastolic Ca^2+^ leak and attenuating the frequency of spontaneous Ca^2+^ transients in aged atrial murine myocytes (Guo et al., 2014), which is an important finding given that aging is associated with an increase risk of AF. Finally, CaMKII is also activated in HF and has been linked to HF-associated arrhythmias. In hypertrophic mice, KN-93 suppressed isoproterenol-induced ventricular arrhythmias and reduced isoproterenol-induced QT_c_ prolongation (Feng et al., 2017). Furthermore, KN-93 significantly reduced arrhythmia inducibility and slowed initiation of ventricular tachycardia in a rabbit model of HF (Hoeker et al., 2016), strengthening the notion that CaMKII inhibition may have antiarrhythmic effects in the failing human heart.

W-7 has been identified as a CaMKII inhibitor; as a CaM antagonist it inhibits the Ca^2+^/CaM dependent activation of CaMKII thereby acting as an upstream blocker of CaMKII (Mazur et al., 1999). In a mouse model of cardiac hypertrophy (in which CaMKII expression levels were increased), inhibition of CaMKII activation by W-7 and KN-93 suppressed polymorphic ventricular arrhythmias (Figure 3). Here, W-7 was found to prolong AP duration to a greater extent than that of KN-93 (Kirchhof et al., 2004). W-7 has been shown to suppress TdP induction, without affecting QT duration or heart rate (Gbadebo et al., 2002; Pu et al., 2005). In a canine model of chronic AV block (associated with compensated hypertrophy and an increased risk of TdP) W-7 suppressed drug-induced TdP and shortened AP duration. Additional *in vitro* experiments found W-7 attenuated the dofetilide-induced enhanced CaMKII phosphorylation (Bourgonje et al., 2012).

#### TRP

Transient receptor potential channels may prove to be suitable therapeutic targets for arrhythmic disorders as a number of these channels have been implicated in arrhythmogenesis and they exhibit specific expression patterns in the cardiac conduction system (Hof et al., 2019). Within the SAN, TRP channels have been identified to contribute to both the membrane and Ca^2+^ clock (Hof et al., 2019). TRPC isoforms 1, 2, 3, 4, 6 and 7 have been detected in the mouse SAN, and treatment with the broad TRP channel inhibitor SKF-96365 results in reduced Ca^2+^ influx and a reduction in the spontaneous pacemaker rate (Ju et al., 2007). Store-operated Ca^2+^ entry (SOCE) describes the influx of Ca^2+^ into the cell when Ca^2+^ levels within the SR become depleted, a process which has been implicated in arrhythmia development (Bonilla et al., 2019). TRPC channels have been identified at store-operated transmembrane Ca^2+^ channels (Bonilla et al., 2019). With TRPC3 being the only TRPC channel to be located at the plasma membrane of pacemaker cells, it has been suggested that this channel is the main isoform to be involved in SOCE in the SAN (Hof et al., 2019). Spontaneous AP events via SOCE-activation were found to be reduced in a TRPC3 knockout mouse model (Ju et al., 2015), while excessive activation of TRPC3 has been shown to result in Ca^2+^ overload and arrhythmias. Under normal conditions, TRPC3 is understood to mediate Ca^2+^ and Na^+^ entry near NCX1, thereby promoting the forward mode of NCX and driving diastolic depolarization (Doleschal et al., 2015; Hof et al., 2019). However, when TRPC activation is increased, Ca^2+^ overload and spatial uncoupling between TRPC3 and NCX1 occurs, resulting in arrhythmogenesis (Doleschal et al., 2015). This evidence therefore suggests that TRPC3 contributes to SAN pacemaking through the stimulation of the Ca^2+^ clock via SOCE, activation of CaMKII and phosphorylation of Ca^2+^ handling proteins (including PLN and RyR2), and via TRPC3-NCX dynamics (Hof et al., 2019).

TRPC3 expression is upregulated in the atria of AF patients, and in numerous animal models of AF (Harada et al., 2012; Zhao et al., 2013), conversely, when TRPC3 expression is deleted, in a TRPC3 knockout mouse model, pacing-induced AF was significantly reduced (Ju et al., 2015). Further studies have suggested TRPC3 overexpression in AF mediates cardiac fibroblast proliferation and differentiation via the regulation of Ca^2+^ influx and downstream Ca^2+^-mediated signaling (Harada et al., 2012). In a canine model of AF, treatment with the selective TRPC3 blocker Pyr-3 reduced atrial fibroblast proliferation and therefore the development of an AF substrate (Figure 4) (Harada et al., 2012). TRPC3 has also been linked to other arrhythmia conditions in addition to AF. Its activation by GSK1702934A has been shown to influence cardiac contractility and favor rhythmic instability (Doleschal et al., 2015), and in a TRPC3 overexpression mouse model, GSK1702934A resulted in arrhythmic events including paired ventricular beats, and episodes of atrial tachycardia, ventricular tachycardia and cardiac alternans (Doleschal et al., 2015). TRPC3 overexpressing hearts were also more susceptible to ANG-II-stimulated arrhythmic events which included atrial or ventricular tachycardia. The interaction between TRPC3 and NCX1 is believed to drive the increased contractility and arrhythmogenesis upon TRPC3 activation (Doleschal et al., 2015). Therefore, inhibiting TRPC3 may be a potential therapeutic approach for arrhythmias arising due to sinus node dysfunction and AF. Pyr-3 has been identified as a selective inhibitor of TRPC3 and has been shown to efficiently suppress physiological events associated with TRPC3, including atrial fibroblast proliferation as highlighted above (Kiyonaka et al., 2009; Harada et al., 2012).

Studies have identified TRPM4 to be involved in SAN pacemaking through modulation of diastolic depolarization, suggesting that therapeutically targeting TRPM4 channels may be useful for heart rate modulation (Hof et al., 2019). Inhibition of TRPM4 by 9-phenanthrol reduced spontaneous AP in isolated mouse and rat atrial preparations and resulted in a frequency-dependent reduction of right atria beating rate (Figure 4 and Table 2). Similar results were evident in rabbit SAN pacemaker cells where inhibition of TRPM4 reduced the diastolic depolarization slope (Hof et al., 2013). The study suggested that TRPM4 may act as a rate accelerator channel in the SAN that becomes activated when heart rate decreases to provide protection against bradycardia (Hof et al., 2013). In addition to TRPM4, TRPM7 has also been detected in the mouse SAN (Hof et al., 2019) and is required for maintaining cardiac automaticity. Deletion of TRPM7 *in vitro* slows spontaneous Ca^2+^ transient frequency and disrupts cardiac automaticity, while *in vivo* a loss-of-function zebrafish model displayed a slowing of heart rate (Sah et al., 2013b). In addition, the study of two mouse models of TRPM7 deletion has revealed a role for TRPM7 SAN function: (1) a cardiomyocyte-specific deletion of TRPM7 resulted in sinus pauses and (2) an inducible SAN/AVN TRPM7 knock-out drove atrioventricular block. These phenotypes were associated with a reduction in diastolic depolarization due to a downregulation of HCN4, and *I*_f_, as a result of TRPM7 deletion (Sah et al., 2013b).

Both TRPM4 and TRPM7 are also expressed in human atrial cells (Hof et al., 2019). Small animal studies have identified that these TRP channels contribute to the atrial AP (Simard et al., 2013), with inhibition of TRPM4 by 9-phenanthrol leading to a shortening of the atrial AP duration, suggesting it may be a potential target for pharmacological approaches against atrial arrhythmias (Simard et al., 2013). It is proposed that TRPM7 channels are involved in fibrogenesis in human AF, and TRPM7 protein expression is upregulated in atrial specimens from AF patients (Zhang et al., 2012). In terms of ventricle conduction, studies using a knockout TRPM4 mouse model found that its loss increased β-adrenergic inotropic response, with TRPM4^–/–^ ventricular myocytes displaying a faster repolarization phase and a shorter AP waveform compared to controls (Mathar et al., 2014). The study concluded that TRPM4 reduces the driving force of Ca^2+^ influx, and subsequently contractile force, with the action of TRPM4 specifically important during β-adrenergic stimulation when there is an increase in the Ca^2+^ current. TRPM4 channels have also been identified in Purkinje fibers, with mRNA expression most abundant in the Purkinje fibers compared to the atria, ventricle and septum (Hof et al., 2019). Inhibition of TRPM4 by 9-phenanthrol was identified to shorten the AP of Purkinje fibers isolated from rabbit hearts with no effect on ventricle AP, suggesting TRPM4 is specifically involved in Purkinje fiber AP regulation (Hof et al., 2016).

The clinical importance of TRPM4 in cardiac conduction has been highlighted by studies that have identified TRPM4 mutations in numerous arrhythmic conditions including progressive cardiac bundle branch disease (Kruse et al., 2009), Brugada syndrome (Liu et al., 2013), LQT syndrome (Hof et al., 2017), atrioventricular and right bundle branch block (Stallmeyer et al., 2012), chronic heart block (Bianchi et al., 2018), and ventricular fibrillation (Bianchi et al., 2018). Furthermore, the cardiac phenotype of TRPM4 knockout mice included conduction block with PR ad QRS lengthening and Luciani-Wenckebach atrioventricular block (Demion et al., 2014). Interestingly, the mutations identified in familial arrhythmic conditions included loss- and gain- of function mutations, which suggests targets may have to be specific for the underlying mutation.

As highlighted in the studies above, 9-phenanthrol is a commonly used inhibitor of TRPM4 (Figure 4 and Table 2). The phenanthrene-derivative has been shown to be a specific inhibitor of TRPM4 channels in numerous tissues in the body (Guinamard et al., 2014). Within cardiomyocytes it is worth noting that 9-phenanthrol has been shown to modulate intracellular Ca^2+^ oscillation in HL-1 cells derived from mouse atrial cardiomyocytes, resulting in a transient increase in intracellular Ca^2+^ levels (most likely from mitochondrial Ca^2+^ stores). The study suggested this may be due to a feedback mechanism from inhibition of TRPM4 since it is known to be activated by Ca^2+^ (Burt et al., 2013). While 9-phenanthrol is a strong TRPM4 inhibitor for *in vitro* and *ex vivo* studies, the use of the agent *in vivo* is limited due low solubility and possible toxicity (Guinamard et al., 2014). In terms of TRPM7, pharmacological agents driving specific TRPM7 inhibition within the heart are lacking. TRPM7 is also one of several TRP channels which display broad tissue expression, suggesting targeting of this channel may result in off-target effects (Hof et al., 2019). Despite this, TRPM7 remains a potential therapeutic target for a range of conditions, including cardiac arrhythmias.

In addition to TRPC and TRPM channels, further TRP channels have been implicated in cardiac rhythm. Increased gene expression of various TRP channels (including TRPM, TRPC, TRPV, and TRPP) have been identified in leukocytes of patients with non-valvular AF (Duzen et al., 2017). TRPV channels have been linked to atrial inotropy related to ROS-mediated signaling (Odnoshivkina et al., 2015), and in a porcine model, depletion of TRPV1 afferent fibers resulted in a reduction in ventricular arrhythmic events associated with MI (Yoshie et al., 2020). A study has shown that TRPA1 may be a novel therapeutic target for increasing inotropic and lusitropic states in the heart, having demonstrated that stimulation of TRPA1 in isolated murine cardiomyocytes resulted in activation of CaMKII-dependent signaling and an increase in peak intracellular Ca^2+^ levels, driving an increase in cardiomyocyte contractile function (Andrei et al., 2017). Finally, in a zebrafish model, mutations in PKD2 (which encodes for TRPP2) resulted in atrioventricular block, with isolated hearts displaying impaired intracellular Ca^2+^ cycling and Ca^2+^ alternans (Paavola et al., 2013).

#### MCU

It has been suggested that cardiac mitochondria influence arrhythmia development through the regulation of cytoplasmic Ca^2+^ fluxes achieved via the uptake of Ca^2+^ through MCU (Brown and O’Rourke, 2010). Much of the work focused on targeting MCU as a potential therapeutic approach to arrhythmogenesis has focused on HF models. A study utilizing a knockout MCU mouse model found, in a non-ischemic HF setting, MCU knockout mice display a shorter AP duration and fewer incidences of EADs and ventricular fibrillation when compared to control animals (Xie et al., 2018). Use of the MCU blocker RR was found to reduce the incidence of ventricular fibrillation in an I/R rat model (Figure 4 and Table 2) (de et al., 2006). However, RR has off-target effects related to Ca^2+^ dynamics, including SR Ca^2+^ release and LTCC (Brown and O’Rourke, 2010). Ru_360_, an RR analog, has been shown to be a more specific MCU inhibitor and able to suppress ventricular fibrillation associated with I/R (Figure 4 and Table 2) (de et al., 2006). Both Ruthenium compounds have also been shown to convert pacing-induced ventricular fibrillation to ventricular tachycardia in isolated rat hearts, highlighting the role of MCU in arrhythmia development (Kawahara et al., 2003). It is worth noting that while Ru_360_ had no effect on left ventricle pressure, at high concentrations RR reduced left ventricle pressure amplitude (Kawahara et al., 2003).

## Discussion

Ca^2+^ plays a critical role in maintaining cardiac physiology and changes to the proteins which are involved in Ca^2+^ handling underly numerous cardiac diseases. Targeting Ca^2+^ dysregulation in HF and arrhythmias has long been investigated as a potential therapeutic target and, as is evident from this review, there is considerable current research investigating targeting proteins associated with Ca^2+^ handling, including RyR2, NCX, SERCA, and TRP channels, as a therapeutic approach for cardiomyopathy, HF and arrhythmias. The pathogenic remodeling of these Ca^2+^ handling proteins has been shown to differ dependent on the underlying pathological mechanism, suggesting a “personalized” approach to targeting Ca^2+^ handling proteins may be required for specific cardiac conditions. Furthermore, while several therapeutics targeting Ca^2+^ have progressed to clinical trials, several are still in early preclinical stages, demonstrating the need for further research in this area. Altogether, targeting Ca^2+^ handling is a promising approach to developing novel and effective therapies for key cardiovascular disorders.

## Author Contributions

EC, CW, and AN contributed conceptually to the manuscript. AN and CW drafted the manuscript. AN contributed the section concerning cardiac hypertrophy and heart failure, and the tables. CW contributed the section regarding cardiac arrhythmias. Both AN and CW contributed to the introduction, the current therapeutic approaches, the discussion, the figures, and the abstract. All authors reviewed the manuscript prior to submission.

## Conflict of Interest

The authors declare that the research was conducted in the absence of any commercial or financial relationships that could be construed as a potential conflict of interest.

## Abbreviations

AC: adenylyl cyclase
AC6: adenylyl cyclase 6
ACE: angiotensin-converting enzyme
ACEI: ACE inhibitors
AF: atrial fibrillation
ANG-II: angiotensin II
AP: action potential
ARB: angiotensin receptor blocker
AVN: atrioventricular node
Ca^2+^: calcium
CaM: calmodulin
CaMKII: Ca^2+^/calmodulin-dependent protein kinase
CPVT: catecholaminergic polymorphic ventricular tachycardia
CVD: cardiovascular disease
DAD: delayed after depolarization
EAD: early after depolarization
ECC: excitation contraction coupling
GWAS: genome wide association studies
HF: heart failure
HFmEF: HF with mid-range ejection fraction
HFpEF: HF with preserved ejection fraction
HFrEF: HF with reduced ejection fraction
HNO/NO: nitroxyl
*I*_Ca_: Ca^2+^ current
*I*_CaL_: L-type Ca^2+^ current
*I*_K_: K^+^ current
*I*_Na_: Na^+^ current
*I*_NCX_: NCX current
*I*_to_: transient outward K^+^ current
I/R: ischemia/reperfusion
LQTS: long QT syndrome
LTCC: L-type Ca^2+^ channel
MAPK: mitogen activated protein kinase
MCU: mitochondrial Ca^2+^ uniporter
MI: myocardial infarction
NCX: Na^+^/Ca^2+^ exchanger
NFAT: nuclear factor of activated T-cell
PKA: protein kinase A
PLN: phospholamban
PMCA: plasma membrane Ca^2+^ ATPase
Pyr-3: pyrazole-3
RAAS: renin–angiotensin–aldosterone signaling
ROS: reactive oxygen species
RR: ruthenium red
RyR: ryanodine receptor
SAN: sino-atrial node
SERCA: SR Ca^2+^ ATPase
SNPs: single nucleotide polymorphisms
SR: sarcoplasmic reticulum
TAC: transverse aortic constriction
TdP: Torsades de Pointes
TRIC: trimeric intracellular cation.
